# Systematic review of outcome domains and instruments used in clinical trials of tinnitus treatments in adults

**DOI:** 10.1186/s13063-016-1399-9

**Published:** 2016-06-01

**Authors:** Deborah A. Hall, Haula Haider, Agnieszka J. Szczepek, Pia Lau, Sarah Rabau, Julie Jones-Diette, Alain Londero, Niklas K. Edvall, Christopher R. Cederroth, Marzena Mielczarek, Thomas Fuller, Angel Batuecas-Caletrio, Petra Brueggemen, Dean M. Thompson, Arnaud Norena, Rilana F. F. Cima, Rajnikant L. Mehta, Birgit Mazurek

**Affiliations:** National Institute for Health Research (NIHR) Nottingham Hearing Biomedical Research Unit, Ropewalk House, 113 The Ropewalk, Nottingham, NG1 5DU UK; Otology and Hearing Group, Division of Clinical Neuroscience, School of Medicine, University of Nottingham, Nottingham, NG7 2UH UK; ENT Department of Hospital Cuf Infante Santo – Nova Medical School, Travessa do Castro 3, 1350-070 Lisbon, Portugal; Department of Otorhinolaryngology, Charite University Hospital, Chariteplatz 1, 10117 Berlin, Germany; Institute of Biomagnetism and Biosignalanalysis, University Hospital Münster, Malmedyweg 15, 48149 Münster, Germany; Department of Otorhinolaryngology and Head and Neck Surgery, University Hospital of Antwerp, Wilrijkstraat 10, 2650 Edegem, Belgium; Centre for Reviews and Dissemination, University of York, York, YO10 5DD UK; Service ORL et CCF, Consultation Acouphène et Hyperacousie, Hôpital Européen G. Pompidou, 20, rue Leblanc, 75015 Paris, France; Experimental Audiology, Department of Physiology and Pharmacology, Karolinska Institutet, Von Eulers väg 8, 171 77 Stockholm, Sweden; Department of Otolaryngology, Laryngological Oncology, Audiology and Phoniatrics, |Medical University of Lodz, 90-549 Lodz, 113 Zeromskiego Street, Lodz, Poland; Clinical Psychological Science, Faculty of Psychology and Neuroscience, Maastricht University, Universiteitssingel 40, PO Box 616, 6200 MD Maastricht, The Netherlands; Adelante, Centre of Expertise in Rehabilitation and Audiology, Zandbergsweg 111, 6432 CC Hoensbroek, The Netherlands; Department of Otorhinolaryngology, IBSAL, University Hospital of Salamanca, Paseo San Vicente 58-182, 37007 Salamanca, Spain; Tinnitus Center, Charite University Hospital, Chariteplatz 1, 10117 Berlin, Germany; Laboratory of Adaptive and Integrative Neuroscience, Centre National de la Recherche Scientifique, Fédération de Recherche 3C, Aix-Marseille Université, Marseille, France

**Keywords:** Adult otolaryngology, Audiology, Clinical trials, Methods

## Abstract

**Background:**

There is no evidence-based guidance to facilitate design decisions for confirmatory trials or systematic reviews investigating treatment efficacy for adults with tinnitus. This systematic review therefore seeks to ascertain the current status of trial designs by identifying and evaluating the reporting of outcome domains and instruments in the treatment of adults with tinnitus.

**Methods:**

Records were identified by searching PubMed, EMBASE CINAHL, EBSCO, and CENTRAL clinical trial registries (ClinicalTrials.gov, ISRCTN, ICTRP) and the Cochrane Database of Systematic Reviews. Eligible records were those published from 1 July 2006 to 12 March 2015. Included studies were those reporting adults aged 18 years or older who reported tinnitus as a primary complaint, and who were enrolled into a randomised controlled trial, a before and after study, a non-randomised controlled trial, a case-controlled study or a cohort study, and written in English. Studies with fewer than 20 participants were excluded.

**Results:**

Two hundred and twenty-eight studies were included. Thirty-five different primary outcome domains were identified spanning seven categories (tinnitus percept, impact of tinnitus, co-occurring complaints, quality of life, body structures and function, treatment-related outcomes and unclear or not specified). Over half the studies (55 %) did not clearly define the complaint of interest. Tinnitus loudness was the domain most often reported (14 %), followed by tinnitus distress (7 %). Seventy-eight different primary outcome instruments were identified. Instruments assessing multiple attributes of the impact of tinnitus were most common (34 %). Overall, 24 different patient-reported tools were used, predominantly the Tinnitus Handicap Inventory (15 %). Loudness was measured in diverse ways including a numerical rating scale (8 %), loudness matching (4 %), minimum masking level (1 %) and loudness discomfort level (1 %). Ten percent of studies did not clearly report the instrument used.

**Conclusions:**

Our findings indicate poor appreciation of the basic principles of good trial design, particularly the importance of specifying *what* aspect of therapeutic benefit is the main outcome. No single outcome was reported in all studies and there was a broad diversity of outcome instruments.

**PROSPERO registration:**

The systematic review protocol is registered on PROSPERO (International Prospective Register of Systematic Reviews): CRD42015017525. Registered on 12 March 2015 revised on 15 March 2016.

**Electronic supplementary material:**

The online version of this article (doi:10.1186/s13063-016-1399-9) contains supplementary material, which is available to authorized users.

## Background

Tinnitus is an auditory percept – often described as a ‘ringing in the ears’– in the absence of a corresponding auditory stimulus and is experienced by approximately 10–20 % of the population [1]. As a symptom there is a no consensus on its aetiology [2, 3] and work is ongoing to profile tinnitus so that interventions can be more specifically targeted [4]. For a subset of individuals, tinnitus severely interferes with activities of daily life, but its impact is wide-ranging and heterogeneous across individuals. Patients report problems in getting to sleep, the need to avoid noisy situations, hearing difficulties, difficulties with concentration, and experience despair, frustration, irritation, depression, fear and worry [5]. Currently, no cure exists for tinnitus but many interventions are being tested [6]. There is reasonable evidence to suggest that cognitive behavioural-based psychological treatments are effective at improving quality of life [7], negative mood, dysfunctional beliefs and tinnitus-related fear [8].

Despite some optimism for treating tinnitus-related distress [9] the field is plagued by a number of fundamental and recurring problems that limit the evidence base and ultimately affect patient care and policy-related decisions. From a trialists’ perspective there is disagreement on what tinnitus-related problems constitute distinct elements of tinnitus, such as perceived loudness or emotional distress, and which are sufficiently important to be considered as domains that should be measured in all studies [10]. This situation has contributed to the high level of diversity in, for example, trial design and measurement of outcomes in confirmatory randomised controlled trials, which hinders comparison and meta-analysis across studies [6]. A recent systematic review examined outcomes of randomised controlled trials of interventions for adults with tinnitus up to March 2013 [11]. However, the review was not concerned with evaluating what was measured, nor the choice of outcome instruments. Rather, it focused on evidence for treatment-related benefits and harms, using this information to develop a clinical practice guideline [12]. Hence, further investigation is warranted to determine more generally what outcomes (namely domains and instruments) are being used in trials of tinnitus interventions.

The difficulties in synthesising evidence from tinnitus trials has negative implications for the provision of effective clinical care since clinicians, insurers, healthcare commissioners, regulatory bodies and other policymakers cannot make informed decisions without good evidence. There are very few practice guidelines and so in the UK and other countries care is not delivered to tinnitus patients in a standardised way [13]. Rather it tends to be driven by reimbursement policies and by which clinical profession (general practitioner, ENT specialist, audiologist, clinical psychologist, etc.) delivers the care.

In sum, the variations in research and in clinical methodologies used to assess, treat, and study tinnitus form a problematic circle, where an incomplete evidence base means that clinical guidelines are developed with limited knowledge, and the lack of standardised clinical practices cannot reliably feed back into addressing important research questions. This scenario is ultimately likely to contribute to an inefficient use of scarce healthcare resources and unnecessary suffering for patients. At present we attempt to break this circle by examining what outcome domains have been defined, and what outcome measures have been used in studies of treatments for adults with tinnitus, by means of a systematic review of publicly available trial protocols. This should ultimately lead to a description of a minimum standard for trialists to choose outcome measures for use in clinical trials that evaluate a tinnitus intervention [10]. A core set would enable results to be more easily compared and synthesised and the most effective interventions to be identified [14].

### Objectives

The primary objective of this systematic review is to identify and evaluate the current reported outcome domains in clinical and experimental studies of adults with tinnitus, with a focus on trial designs investigating the treatment of tinnitus, and published between the date of an international consensus meeting in July 2006 [15] and March 2015. Data collection considered both which domain of tinnitus was identified as important for demonstrating therapeutic benefit and which instrument was used to assess that domain. Three secondary objectives considered the choice of instruments with respect to identifying patterns: (1) across continents to determine whether there are geographical preferences for using one primary outcome instrument over another, (2) across years to determine changes over time in the uptake of outcome instruments as a primary outcome, and (3) across interventions to determine whether particular classes of intervention favour using one primary outcome instrument over another.

## Methods

Details of the study eligibility criteria, information sources, search strategy, selection and data collection processes, as well as data synthesis methods were published as a protocol in advance of completing the data collection [16]. Reporting is guided by the Preferred Reporting Items for Systematic reviews and Meta-analyses (PRISMA) [17] and are described using the PRISMA checklist (see Additional file 1).

### Eligibility criteria

Study eligibility was defined according to PICOS (Patient, Intervention, Comparison, Outcome, Setting) and there were no modifications to the published protocol [16]. All included studies assessed adults (men and women) aged 18 years or older who reported tinnitus as one of their primary complaints, irrespective of whether they were recruited from clinical or non-clinical populations. There were no restrictions on the type of intervention as long as the main motivation was to bring about a therapeutic benefit for people with tinnitus. Studies in which the impact on tinnitus was of secondary relevance (e.g. where reducing hearing problems was the primary aim) were excluded. Consistent with this approach, only those studies reporting tinnitus-related changes as a primary outcome were included, irrespective of how those changes were measured. The systematic review included randomised controlled trials, before and after studies, non-randomised controlled trials, case-controlled studies and cohort studies. There were no restrictions on research settings.

To be included in this report, articles were required to be written in English and published in or after July 2006 [15]. These decisions were motivated by resource limitations. Furthermore, to improve clinical and scientific value, any studies either recruiting fewer than 20 participants with tinnitus or having fewer than 20 at the end point of the study were excluded. This cut-off was selected in advance, following Needleman et al. [18]. We included published systematic reviews and meta-analyses that considered tinnitus trials meeting the above criteria. These reviews and meta-analyses were not subject to the data collection process itself, but we did a hand-search and include any additional eligible studies reported within them.

During the data collection process, a small number of studies were identified where age-related eligibility or target sample size were missing. In cases where neither pieces of information were reported, the corresponding author was contacted for more details by email, with one reminder.

### Information sources

Studies were identified by searching electronic databases of research literature (Table 1). The following list details the database, as well as the number of records identified by the search strategy (in parentheses): PubMed (National Centre for Biotechnology Information) (*n* = 759), EMBASE (Ovid) (*n* = 244), Cumulative Index to Nursing and Allied Health Literature (CINAHL, EBSCO) (*n* = 145) and the Cochrane Central Register of Controlled Trials (CENTRAL) (*n* = 560). A number of different electronic trial registers were also searched: ClinicalTrials.gov (*n* = 141), the International Standard Randomised Controlled Trial Number registry (ISRCTN, BioMed Central) (*n* = 22), the International Clinical Trials Registry Platform (ICTRP, World Health Organisation) (*n* = 183), and the Cochrane Database of Systematic Reviews (CDSR) (*n* = 23). Electronic searches were run on 12 and 13 March 2015 by authors DAH and AJS, and were not updated.

**Table 1:** Table summarising the electronic information sources used. For a description of the abbreviations, see text

Type of electronic search	Database	Number of items (*n*)
Academic databases	PubMed	759
	EMBASE	244
	CINAHL	145
	CENTRAL	560
Clinical trial registers	ClinicalTrials.gov	141
	ISRCTN	22
	ICTRP	183
	CDSR	23

In addition, a hand-search was conducted using the 251 published records that had met eligibility at the abstracts and full-text screening stages. Specifically, we hand-searched the set of registered clinical trials to identify any further registers of the same trial and also to identify any published protocols or study findings that were indexed to that trial by its unique study identifier. We also manually searched the 18 systematic review articles to look for any overlooked studies for inclusion. An additional 52 records were identified by these approaches. Following this step, the systematic review articles themselves were not included for data collection purposes.

### Search strategy

The search strategy used in this systematic review was previously published [16]. Search terms for PubMed, EMBASE, and CINAHL were informed by the PICOS criteria and were: (1) tinnitus AND (2) stud* OR clinical trial* OR therap* OR treatment* OR intervention*. Where possible the search was limited to humans (not animals), adults (not paediatric), English language and 2006-date of search. The syntax for the subsequent search of the CENTRAL trials registry of the Cochrane Collaboration was: #1 tinnitus; #2 Paediatric:TI,AB,KY; #3 Pediatric:TI,AB,KY; #4 child*:TI,AB,KY; #5 #1 NOT #2 NOT #3 NOT #4, #6 english:LA, #7 #5 AND #6, #8 (2006–2015):PD NOT IN MEDLINE NOT IN EMBASE AND 2006 TO 2015:YR, and #9 #5 NOT INMEDLINE NOT INEMBASE. Electronic trial registers all used ‘tinnitus’ as the main search term.

### Data management

DAH was responsible for data management and maintained the editorial rights. All identified records were saved into a Microsoft Excel master file where records were tracked through the screening and data collection process by a unique study identification code. A simple system of record annotation was implemented to capture reasons for exclusion. At the end of data collection, checking and formatting, a pdf copy of the master file was created as a ‘locked’ record so that there is a version of the data that cannot be edited in error (7 December 2015). An editable Excel version of this document can be downloaded (see Additional file 2).

### Selection process

**Fig. 1:**
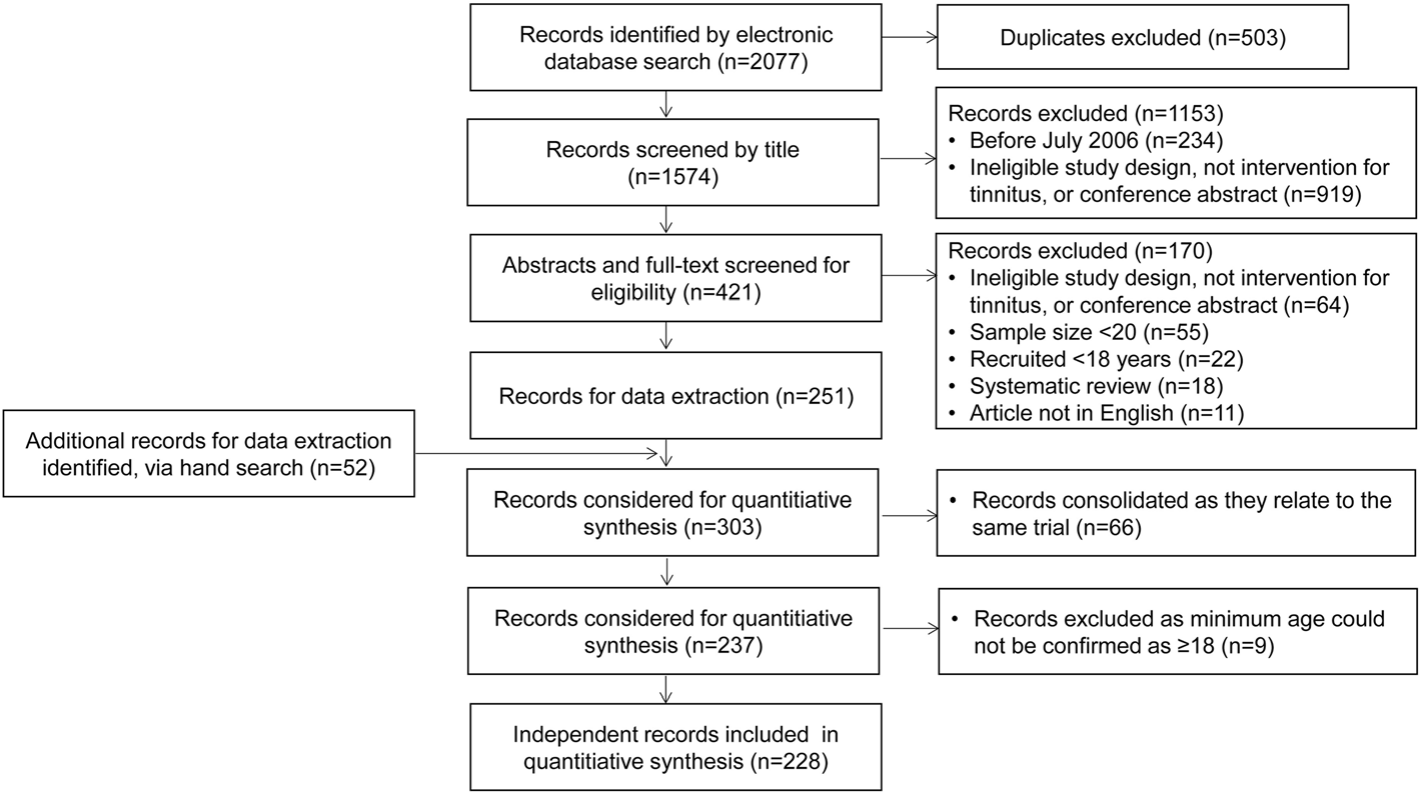
Flow diagram of study records

Endnote was used to remove 141 duplicate records from the PubMed, EMBASE and CINAHL searches, while the remaining 362 duplicates were manually identified within the Excel master file by DAH and HH using author names, study title and trial registration number. This gave a total of 1574 records for eligibility screening. Screening steps were carried out DAH, HH and AJS. Following the pre-specified protocol, a two-step process was implemented to decide eligibility: first by reading the title, and second by reading the abstract and full text. It was possible to exclude 1153 records by title and summary information alone (see Fig. 1). Full texts were obtained for the 421 remaining records that potentially met the inclusion criteria or for which there was insufficient summary information to make a clear decision. From this step, a further 170 records were excluded, leaving 251 for data extraction. It is interesting to note that almost one third of those records excluded at this step was due to the small sample size of the study (see Fig. 1). Twenty-two records were excluded because they recruited participants below 18 years of age. Moreover, 55 full texts were excluded because the sample size was less than 20 participants and 11 full texts were excluded because they were not available in English. Instead, these were published in national journals written in the native language. So that the reader can scrutinise the data for evidence of geographical bias in these three full-text exclusion criteria, details are broken down by country in Table 2. This information gives some indication for a risk of bias excluding tinnitus studies conducted in the USA since 21 were removed on the basis of small sample size, leaving only 39 records from the USA contributing to the systematic review. There is also a risk of bias excluding tinnitus studies conducted in China since six were removed because they were published in Chinese, leaving only three records from China contributing to the systematic review. Note that language bias was avoided for studies registered on ISRCTN and ICTRP since an English language translation is given. Ten trials in Iran, seven in Japan and two in China, two in Brazil and one in the Republic of Korea were included via this route.

**Table 2:** Summary of those records excluded at the full-text screening stage because (1) the sample size was less than 20 participants, (2) because the articles were not available in English, or (3) they recruited participants below 18 years of age. Details are broken down by country

	Sample size <20	Non- English language	Minimum eligibility (age in years)				
			13	14	15	16	17
Austria				1			1
Belgium	2					1	
Czech Republic							
Finland	2						
France	3						
Germany	4	1		2		4	
Italy	3						1
Spain	1						
Sweden	3						
Switzerland	1					1	
The Netherlands	3						
Turkey					1		
Iran		2			1		
Iraq			1				
Israel	1						
Egypt				1			
Brazil	2	1			1		
Uruguay	1						
China		6			1		1
Japan	3						
Republic of Korea	1	1		1		1	
Australia	2						1
New Zealand	1					1	
USA/Canada	22						

At least two co-authors performed each key step (i.e. title screening, full-text screening, and data collection) independently for every record. Due to an error in allocating full texts to co-authors, some records had data collection by more than two co-authors (31 were completed by three co-authors, 11 by four and 9 by five). Discrepancies between independent co-authors were rare and were mostly accountable by differences in terminology. These were resolved by DAH who was responsible for data management. As per the protocol [16], inter-rater agreement was not calculated, but all co-authors reviewed and approved the master file before data lock.

We pieced together data from multiple reports of the same study by manually screening all included records using author names, study title and trial registration number. This step of consolidating records happened throughout the data collection process, and in particular during the data formatting check. Where there were multiple reports, the data extraction reflects the information provided in the report with the latest publication date. Any discrepancies between information reported in the different articles were noted under the data item heading ‘intention versus reporting’.

### Data collection process

We contacted 29 trialists to request missing information about the minimum age for inclusion and two investigators to request missing information about sample size. With respect to age, 20 confirmed that all participants were 18 years of age or older, two authors could no longer be contacted, two responded but were unable to confirm the minimum age, one responded but said he was too busy to provide the information, and four did not respond. On the basis of this, nine records were excluded. Both investigators who were contacted about sample size were able to provide the required information and so these records were included. A summary of those relevant records are provided in more detail in an additional Table (see Additional file 3). After exclusion, 228 records were included for data collection. A further Table provides full references (see Additional file 4).

Data items gave rise to headings in a data collection sheet. Data collection was guided by an electronic form (Excel spreadsheet) that was also used to collate all responses. Data collection was conducted by a pool of 20 project team members (number of extracted studies ranged from 5 to 228, median 19.5). The primary reason for not limiting data collection to a smaller pool was to lessen the resource burden since we received no grant funding to conduct the research activity. To mitigate against observer bias, a full set of guidance notes was produced for the data collection procedure and calibration exercises were conducted with new members of the review team prior to any individual contribution to this review. Both the sheet and the guidance notes were developed and revised across several review authors during a 3-day workshop and through two iterations of piloting. Data collection was conducted independently and with at least two team members for every included record. In an amendment to the pre-specified protocol, DAH verified the data collection for all included records to ensure consistency in approach and in terminology; the latter being necessary for automated data counting. Another step to mitigate against observer bias during the data collection process was by avoiding any instance where an individual extracted data relating to one of their own trials.

### Data items

Data items included all of the fields reported in the published protocol [16]. A majority of data items fall within the PICOS framework. Participant data items relating to the inclusion criteria for each trial record were: (1) minimum age, (2) maximum age (if any), (3) tinnitus duration, (4) intermittent or constant tinnitus, (5) pulsatile or non-pulsatile tinnitus, (6) tinnitus severity, (7) any other subtypes of tinnitus, and (8) any other health-related comorbidities. Participant data items relating to the exclusion criteria for each trial record were: (9) any other subtypes of tinnitus, and (10) health-related comorbidities. Intervention data items recorded the (11) type and (12) duration of intervention in each arm of the trial. Data items describing the study design (i.e. ‘comparison’) comprised: (1) a pull-down list of study design options (randomised controlled trials, before and after studies, non-randomised controlled trials or case-control studies and cohort studies) and (2) a record of the duration of each intervention, separately for each arm of the trial. Outcome data items were: (1) the outcome domain(s) specified by the investigators, (2) the instruments specified by the investigators, and (3) time frame. Information relating to these three data items was recorded separately for all primary and secondary outcomes. Where authors were not explicit about this distinction, we tried to tease this information out of the article by reading the Methods and Results sections of each record. But if this was not possible, then all information was entered as a primary data item. A ‘setting’ data item reported the country where the study was conducted. Supplementary information was also extracted from each included trial on: (1) the name and email address of the corresponding author, (2) the date of study start, (3) the aim of the trial, (4) sample size calculation, with a full-text extraction of the reported details, (5) the sample size, (6) a description of any modifications to the methods, particularly any discrepancies between the trial protocol and the subsequent report of the findings, and (7) the date of publication. The protocol was amended so that if minimum age of eligibility or sample size estimate was not reported, then the data collection recorded the minimum age of the recruited participants or the recruited sample size as the ‘next best alternative’, where this information was given. An additional data item not planned in the protocol recorded whether the study authors specified any minimal clinically important difference, or related construct that was used to interpret the clinical significance of the findings. For example, Cima and et al. [8] specified a pre- versus post-intervention change of 0.065 (SD 0.15) in health utility score measured using the 36-item short form Health Survey. This information is not reported here, but will be presented in a separate manuscript. If any information is not reported, then ‘not stated’ was recorded in the corresponding field.

Where a trial record consolidated several pieces of information (such as a protocol and the published findings), the data items reported in the synthesis related to the most recent publication. For those records in which several pieces of information are consolidated into a single record, we sought to detect any modifications to the methods leading to inconsistencies between the protocol and the final reported study. Given that the review focused on the design of clinical trials, wherever possible information relating to each data item was taken from the study design reported in the most recent publication, not from any report of the study results. For example, sample size recorded the estimated sample size not the number of participants actually enrolled into each intervention arm. And, the date of publication recorded the date of the print copy, not the date of first submission, acceptance or the date of 'online first' publication.

### Outcomes and prioritisation

The primary research question in this review concerned the outcome domains (and instruments) being used in clinical trials of tinnitus treatment. Therefore, the priority for data synthesis and reporting of findings was data relating to all primary outcomes. Where authors failed to distinguish between primary and secondary outcomes, we classified them all as primary. Those outcomes explicitly defined as secondary were also examined, but as a secondary research question.

### Risk of bias in individual studies

Given that the primary objective of this systematic review concerns methodology (not therapeutic effects), we limited the assessment of risk of bias to the data collection methods for consolidated records rather than any analysis of those data. In particular, we investigated where there were inconsistencies between the outcomes defined in the trial registration and/or protocol and those given in the subsequent study report. Of the 228 studies selected for inclusion, 60 (26 %) had multiple records. We examined only those consolidated records with a protocol and study report(s) comparing data items across records. From this set, 21 were found to have descriptions of eligibility criteria (inclusion or exclusion), primary outcome measures, and/or secondary outcome measures that were altered retrospectively in the final report. An additional Table gives more details about the findings from the risk of bias assessment (see Additional file 5). None of the studies reported a justification for the changes, but insufficient information was given in the publications to determine any instances of intentional deception (i.e. outcome-reporting bias) where outcomes had been selected on the basis of the results, for inclusion in the publication of trial findings [19–21]. We did not contact authors to examine reasons for altered reporting.

## Results

The primary objective was to identify and evaluate the current reported outcome domains and instruments in designs of intervention studies of adults with tinnitus, published since July 2006.

### Domains

For the first part of the analysis, we scrutinised the data collected under the data item relating to the primary outcome domain(s) specified by each set of investigators. There were 505 data entries describing 35 different types of primary domain (Table 3). Domain grouping was conducted by a subgroup of tinnitus experts (three ENT surgeons, one audio-vestibular physician, and two researchers) and was broadly informed by the Cochrane Effective Practice and Organization of Care (EPOC) recommendations [22]. Patient outcomes concerned with health status, well-being and health behaviours constituted the largest category by far and so we expanded this into domains relating to (1) the tinnitus percept, (2) the impact of tinnitus, (3) other co-occurring complaints, (4) health-related quality of life, and (5) body structures and functions (Table 3). Remaining EPOC categories were (6) adverse events or harms and (7) satisfaction, with further categories for (8) treatment-related outcomes, and (9) for domains that were unclear or not specified by the author. The most popular primary outcome domain directly relating to tinnitus was ‘tinnitus loudness’ (*n* = 70, 14 % defined as primary outcome domain in all studies), with ‘tinnitus distress’ (*n* = 33, 7 %) and ‘tinnitus annoyance’ (*n* = 21, 4 %) following.

**Table 3:** Summary of all primary and secondary outcome domains across all 228 clinical trials. Domains have been grouped according to eight major topic categories. Categories 1–5 relate to different types of ‘patient outcomes’, categories 6–7 relate to ‘adverse events’ and ‘satisfaction’, following the Effective Practice and Organisation of Care classification scheme [22]. Categories 8 and 9 best describe the remaining outcomes reported in the included records. Percentages are rounded so, for example, 0 % denotes a value that is <0.5 %

	Primary domains		Secondary domains	
	Number	Percentage (%)	Number	Percentage (%)
(1) Domains relating to the tinnitus percept:				
Tinnitus loudness	70	14 %	42	7 %
Tinnitus pitch	12	2 %	13	2 %
(2) Domains relating to the impact of tinnitus:				
Tinnitus distress	33	7 %	18	3 %
Tinnitus annoyance	22	4 %	15	3 %
Tinnitus awareness	10	2 %	2	0 %
Cognition	2	0 %	4	1 %
Behaviour	1	0 %	0	-
Acceptance of tinnitus	0	-	3	1 %
Catastrophising	0	-	1	0 %
Concentration	0	-	2	0 %
Tinnitus intrusiveness	0	-	2	0 %
Tinnitus-related cognitions	0	-	1	0 %
Tinnitus-related fear	0	-	1	0 %
(3) Other co-occurring complaints:				
Depression	8	2 %	18	3 %
General distress	5	1 %	5	1 %
Anxiety	4	1 %	6	1 %
Anxiety and depression	4	1 %	13	2 %
Hearing threshold	4	1 %	11	2 %
Hearing loss	2	0 %	1	0 %
Speech perception	2	0 %	0	-
Hearing handicap	1	0 %	3	1 %
Hearing loss annoyance	1	0 %	0	-
Sleep quality	1	0 %	12	2 %
Somatic sensations	1	0 %	1	0 %
Fear (anxiety)	0	-	1	0 %
Hyperacusis	0	-	3	1 %
Mood	0	-	2	0 %
Sound tolerance	0	-	1	0 %
Speech discrimination	0	-	3	1 %
(4) Health-related quality of life (QoL):				
QoL (tinnitus)	16	3 %	13	2 %
QoL	9	2 %	20	3 %
Coping	3	1 %	0	-
Occupational health	0	-	1	0 %
QoL (hearing)	0	-	1	0 %
Sense of control	0	-	1	0 %
(5) Body structures and functions:				
Neck mobility	1	0 %	1	0 %
Neural activity	1	0 %	2	0 %
Oxidative stress	1	0 %	0	-
Active myofascial trigger points	0	-	1	0 %
Blood parameters	0	-	1	0 %
Gene expression	0	-	1	0 %
Metabolism	0	-	4	1 %
Neck pain	0	-	1	0 %
Neuroendocrine hormones	0	-	1	0 %
Pharmacokinetics	0	-	1	0 %
Structural brain change	0	-	1	0 %
(6) Adverse events or harms:				
Safety and tolerability	6	1 %	4	1 %
Safety	2	0 %	43	7 %
Drug safety and tolerability	1	0 %	4	1 %
Side effects	1	0 %	15	3 %
Headache	0	-	1	0 %
Pain frequency	0	-	1	0 %
Pain intensity	0	-	1	0 %
(7) Satisfaction:				
Treatment satisfaction	1	0 %	5	1 %
(8) Treatment-related outcomes:				
Withdrawals	1	0 %	0	-
Adequacy of blinding	0	-	1	0 %
Credibility (sham)	0	-	1	0 %
Credibility (treatment)	0	-	2	0 %
Needling sensation (acupuncture)	0	-	1	0 %
Therapeutic alliance	0	-	1	0 %
Tolerability	0	-	5	1 %
(9) Domain of interest unclear or not specified by the authors:				
Not specified	128	25 %	140	24 %
Cannot code	58	11 %	76	13 %
Multi-domain specification	10	2 %	10	2 %
Tinnitus severity	69	14 %	29	5 %
Tinnitus handicap	14	3 %	5	1 %
Total	505	100 %	579	100 %

Over half (*n* = 279, 55 %) of the data entries did not clearly describe the complaint of interest. Since this was such a large percentage, we chose to examine this in more detail rather than simply report as a quantitative summary of quality, as per the protocol [16]. Instead, we sought to describe the ways in which the authors’ specification of each primary outcome domain appeared to be inadequate using a narrative approach. Primary outcome domains in category 7 were classified into five subheadings (Table 3). On 128 occasions (25 %), the investigators did not explicitly state which domain their trial intended to assess and so we refer to these as ‘not specified’. ‘Tinnitus severity’ was the next most common phrase used to define the outcome domain of interest (*n* = 69, 14 %). We note that in our protocol [16], we had stated that this is not an adequate domain because it does not explain the dimension of complaint on which severity should be considered. The same applies to ‘tinnitus handicap’ (*n* = 14, 3 %). We also experienced difficulty in interpreting a further 58 (12 %) data entries because the terminology was indeterminate (referred to as ‘cannot code’). We are confident that this is not a coding issue, as DAH verified that the data collection for all included records captured the text as reported by the authors. Examples include ‘improvement’, ‘treatment responder’, ‘change’, ‘tinnitus impact’, ‘size of tinnitus problem’, ‘tinnitus impairment’, ‘problems associated with tinnitus’, ‘difficulties due to tinnitus’, ‘degree of tinnitus’, ‘sensation of tinnitus’, and ‘tinnitus characteristics’. Again, none of these clearly explain the dimension of complaint on which improvement or problems should be considered. ‘Multi-domain specification’ refers to composite measures describing several different complaints such as ‘tinnitus annoyance and distress’ and ‘internal thoughts, sensations and feelings’ (*n* = 10, 2 %).

There were 579 data entries describing 60 different types of secondary domain. Again, Table 3 indicates similar patterns, with ‘tinnitus loudness’ (*n* = 42, 7 %), with ‘tinnitus distress’ (*n* = 18, 3 %) and ‘tinnitus annoyance’ (*n* = 15, 3 %) being the most popular. Safety (*n* = 43, 7 %), Quality of life (*n* = 20, 3 %), and depression (*n* = 18, 3 %) were also popular as secondary outcome domains.

### Instruments

The second part of the primary objective was to identify and evaluate the current reported outcome instruments and for this we interrogated the data collected under the data item relating to the primary outcome instrument(s) specified by each set of investigators. Overall, there were 505 data entries describing 78 different types of instrument (Table 4). We used a categorisation scheme based on the one for domains. Instruments were grouped according to whether the tests relate to: (1a) the tinnitus percept (investigator-administered), (1b) the tinnitus percept (numerical rating scale), (2a) the impact of tinnitus (patient-reported questionnaire), (2b) the impact of tinnitus (numerical rating scale), (3) other co-occurring complaints, (4a) health-related quality of life (patient-reported questionnaire), (4b) health-related quality of life (numerical rating scale), (5) body structures and functions, (6) adverse events or harms, (7) satisfaction, (8) treatment-related outcomes, or (9) were unclear or not specified by the authors. Twenty-eight different instruments were used only once as a primary outcome and these are listed in an additional Table (see Additional file 6).

**Table 4:** Summary of all primary and secondary outcome instruments used across all 228 clinical trials. Instruments have been grouped according to the major domain categories reported in Table 3, as well as those instruments that were not clearly specified by the authors. Note that the total refers to the number of instruments across all 228 trials. The remainder are reported in Additional file 6. Percentages are rounded so, for example, 0 % denotes a value that is <0.5 %

	Primary outcome instruments		Secondary outcome instruments	
	Number	Percentage (%)	Number	Percentage (%)
(1a) Investigator-administered tests relating to the tinnitus percept:				
Tinnitus loudness matching	20	4 %	16	3 %
Tinnitus pitch matching	9	2 %	22	4 %
Minimum masking level	5	1 %	12	2 %
Loudness discomfort level	3	1 %	2	0 %
Tinnitus bandwidth matching	0	-	2	0 %
(1b) Patient-reported numerical rating scales relating to the tinnitus percept:				
Tinnitus loudness	37	8 %	24	4 %
Tinnitus pitch	0	-	2	0 %
(2a) Patient-reported questionnaire instruments relating to the impact of tinnitus:				
Tinnitus Handicap Inventory	77	15 %	31	5 %
Tinnitus Questionnaire (German version)	29	6 %	11	2 %
Tinnitus Questionnaire (English version)	5	1 %	0	-
Tinnitus Functional Index	18	4 %	3	1 %
Tinnitus *Beeinträchtigungs Fragebogen*	13	3 %	8	1 %
Tinnitus Severity Index	12	2 %	1	0 %
Tinnitus Reaction Questionnaire	11	2 %	2	0 %
Tinnitus Handicap Questionnaire	8	2 %	5	1 %
Mini-Tinnitus Questionnaire	6	1 %	0	-
Tinnitus Effects Questionnaire	2	0 %	0	-
Tinnitus diary	2	0 %	0	-
Tinnitus Psychological Impact Questionnaire	2	0 %	0	-
Tinnitus Severity Scale	0	-	6	1 %
Tinnitus Acceptance Questionnaire	0	-	6	1 %
(2b) Patient-reported numerical rating scales relating to the impact of tinnitus:				
Tinnitus distress	7	2 %	8	1 %
Tinnitus annoyance	21	4 %	14	2 %
Tinnitus awareness	10	2 %	2	0 %
(3) Patient-reported questionnaire instruments relating to other co-occurring complaints:				
Beck Depression Inventory	7	1 %	13	2 %
Hospital Anxiety and Depression Scale	7	1 %	27	5 %
Perceived Stress Questionnaire	3	1 %	0	-
Spielberger State and Trait Anxiety Inventory	3	1 %	3	1 %
Brief-Coping with Problems Experienced	2	0 %	0	-
Pittsburgh Sleep Quality Index	2	0 %	2	0 %
Hearing Handicap Inventory	1	0 %	4	1 %
Hyperacusis questionnaire (undefined)	1	0 %	2	0 %
Attention and Performance Self-assessment Scale	0	-	2	0 %
* Befindlichkeitsskala*	0	-	2	0 %
Cognitive Failures Questionnaire	0	-	2	0 %
Depression Anxiety and Stress Scale	0	-	3	1 %
Insomnia Severity Index	0	-	7	1 %
Major Depression Inventory	0	-	5	1 %
Sleep Questionnaire (undefined)	0	-	3	1 %
(4a) Patient-reported questionnaire instruments relating to health-related quality of life:				
Clinical Global Impression Scale	4	1 %	14	2 %
36-item short form Health Survey	2	0 %	6	1 %
WHOQOL-BREF	0	-	10	2 %
EuroQoL	0	-	2	0 %
Quality of Life Inventory	0	-	2	0 %
(4b) Patient-reported numerical rating scales relating to health-related quality of life:				
Quality of Life (tinnitus)	5	1 %	6	1 %
Quality of Life	4	1 %	1	0 %
(5) Technical and laboratory measurements relating to body structures and functions:				
Pure tone audiometry	15	3 %	23	4 %
Speech audiometry (various types)	6	1 %	11	2 %
Electroencephalography	4	1 %	5	1 %
Blood chemistry	2	0 %	10	2 %
Positron Emission Tomography	2	0 %	0	-
Electrocardiogram	1	0 %	4	1 %
Digit symbol test	1	0 %	2	0 %
Blood drug levels	0	-	4	1 %
Magnetic Resonance Imaging	0	-	3	1 %
Otological examination	0	-	4	1 %
Otoscopy	0	-	2	0 %
Psychoacoustic assessment (undefined)	0	-	3	1 %
Tympanometry	0	-	2	0 %
Urine analysis	0	-	2	0 %
(6) Measures of adverse events or harms:				
Adverse events/Side effects	4	1 %	30	5 %
(7) Measures of satisfaction:	No instruments reported			
(8) Measurement instruments of treatment-related outcomes:				
Withdrawal rate	2	0 %	2	0 %
(9) Measurement of interest unclear or not specified by the authors:				
Cannot code	20	4 %	52	9 %
Other numerical rating scale (undefined)	18	4 %	29	5 %
Questionnaire (authors’ own)	15	3 %	13	2 %
Numerical rating scale of tinnitus severity	12	2 %	1	0 %
Not specified	8	2 %	18	3 %
Total	505	87 %	579	85 %

Instruments assessing the impact of tinnitus were most common and of these, the Tinnitus Handicap Inventory was the most popular (*n* = 77, 15 %) [23] and was one of the instruments recommended by the 2006 consensus meeting [15]. Other recommended questionnaires were the Tinnitus Questionnaire (*n* = 34, 7 %), the Tinnitus Reaction Questionnaire (*n* = 11, 2 %), and the Tinnitus Handicap Questionnaire (*n* = 8, 2 %). However, our review indicates that the Tinnitus Functional Index, Tinnitus *Beeinträchtigungs Fragebogen* (a shortened version of the Tinnitus Handicap Inventory translated into German) and Tinnitus Severity Index were just as widespread.

Tinnitus loudness matching was a popular tool for assessing the tinnitus percept (*n* = 20, 4 %). A numerical rating scale of loudness was also a common approach (*n* = 37, 8 %), but there was little consistency in the measurement scale used (e.g. Table 5). Other domains relating to the impact of tinnitus were evaluated using a numerical rating scale predominantly annoyance (*n* = 21, 4 %), awareness (*n* = 10, 2 %), and distress (*n* = 7, 1 %). Numerical rating scales with 0–10 and 0–100 point scales were popular.

**Table 5:** Summary of the different formats for numerical rating scales used across all 228 clinical trials. These are used to assess a wide range of domains including tinnitus loudness annoyance, awareness, distress and tinnitus-related quality of life

	Primary outcome instruments		Secondary outcome instruments	
	Number	Percentage (%)	Number	Percentage (%)
Numerical rating scale (0–3)	1	0 %	0	-
Numerical rating scale (0–10)	49	10 %	18	3 %
Numerical rating scale (0–100)	18	4 %	13	2 %
Numerical rating scale (1–9)	0	-	1	0 %
Numerical rating scale (1–10)	12	2 %	0	-
Numerical rating scale (1–100)	1	0 %	0	-
Numerical rating scale (4 points)	3	1 %	2	0 %
Numerical rating scale (5 points)	2	0 %	2	0 %
Numerical rating scale (7 points)	3	1 %	0	-
Numerical rating scale (10 points)	0	-	1	0 %
Numerical rating scale (10-cm line)	5	1 %	14	2 %

About 16 % (*n* = 78) of the data entries did not clearly report the instrument used. These were classified into five subheadings under Table 4, category 9. On 20 occasions (4 %), we experienced difficulty in interpreting the data entry (referred to as ‘cannot code’). One recurring example was where investigators did not state the provenance of the ‘tinnitus questionnaire’ which could be either a published Tinnitus Questionnaire [24, 25], or a translation of one of these or to an authors’ own instrument. We observed 15 instances (3 %) where investigators reported using their own (unpublished) questionnaire, which limits reproducibility.

There were 579 data entries describing 108 different types of secondary instrument (Table 4). Of those, 49 instruments were used only once as a secondary outcome and these are listed separately in a Table (see Additional file 6). Although the Tinnitus Handicap Inventory remained a common choice as a secondary outcome (*n* = 31, 5 %), other tinnitus-related questionnaires were much less so. Instead, adverse events (*n* = 30, 5 %) and the Hospital Anxiety and Depression Scale (*n* = 27, 5 %), pure tone audiometry (*n* = 23, 4 %), tinnitus pitch matching (*n* = 22, 4 %), the Clinical Global Impression Scale (*n* = 14, 2 %) and the WHOQOL-BREF (*n* = 10, 2 %) were some of the more popular choices for secondary outcomes.

The protocol did state that the timing of the primary end point would be examined [16], but we did not pursue this analysis because the timing of the end point was reported inconsistently across studies (some relative to the start of the treatment and others relative to the end of the treatment) and the duration of treatment varied so greatly (some were just a few days, some extended up to 1 year, and others did not clearly specify). Moreover, the time frame of surveillance for adverse events was rarely stated.

### Pattern of primary outcome instruments across world regions

**Fig. 2:**
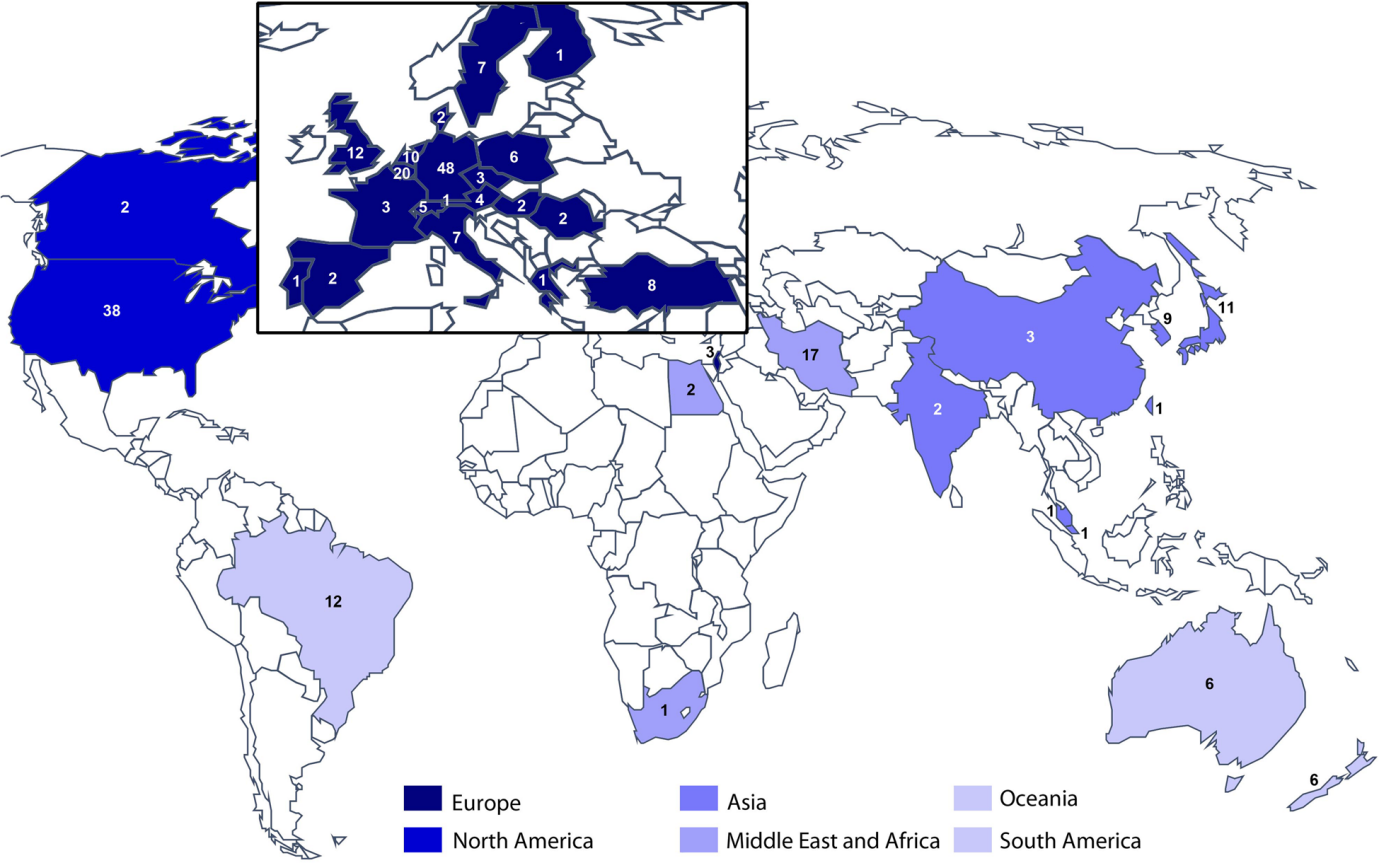
World map illustrating the distribution of recruiting sites for all included studies, inspired by the World Health Organization (WHO) regional classification. Figures within each country indicate only one trial [27] had a recruiting site in South Africa and so this was combined with countries in the WHO Eastern Mediterranean region to create the Middle East and Africa region (*n* = 20). The WHO Region of the Americas was separated into North and South America because we anticipated that language differences might influence choice of outcome instruments. Similarly, Australia and New Zealand were considered separately from Western Pacific region, as Oceania, while other countries were combined with the WHO Southeast Asia region to create a single Asian region

The first secondary analysis assessed how the pattern of primary outcome instruments varied across world regions. Countries recruiting into identified clinical trials were categorised into six world regions using the World Health Organisation (WHO) as a guide [26]. Findings are summarised in Fig. 2. The ‘European region’ represented the greatest research activity with 151 sites recruiting across all 228 trials. Most research was conducted in Germany (*n* = 48), Belgium (*n* = 20), and UK (*n* = 12). In the Middle East and Africa region, most trials were conducted in Iran (*n* = 17), while in Asia most research was conducted in Japan (*n* = 11) and the Republic of Korea (*n* = 9).

With respect to patient-reported questionnaires relating to the impact of tinnitus, the Tinnitus Handicap Inventory was the most common one used as a primary outcome across all world regions, except for Oceania where the Tinnitus Reaction Questionnaire was preferred. Since few clinical trials were conducted in South America or Oceania, findings for these world regions should be interpreted with caution. The Tinnitus Questionnaire was common in Europe, especially in Germany, but not in the rest of the world. Even in Europe, it is used in different forms because the English and German versions differ from one another [28]. The Tinnitus Severity Index was common in the Middle East and Africa region, but not in other parts of the world. Measures of tinnitus loudness were also most common in the Middle East and Africa region (both using loudness matching and numerical rating scales), with countries in Asia also favouring a loudness numerical rating scale.

### Pattern of usage of primary outcome instruments across years

**Fig. 3:**
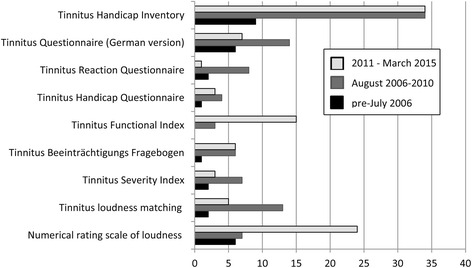
Pattern of usage over time for selected primary outcome instruments. Note that the identification of studies categorised as ‘pre-July 2006’ may not be representative as many records relating to this period would have been excluded according to our search criteria

We also examined the status of selected primary outcome instruments over the time frame of the review (Fig. 3). Due to the wide variety of instruments, analysis focused on the most frequently used that were highlighted in the previous section (Instruments). For meaningful analysis, we split the total time frame into three periods, using the best available information. The first period was from 1 January 2011 to 12 March 2015 (i.e. the date of the electronic searches) (*n* = 102). The second period was from 1 August 2006 to 31 December 2010 (*n* = 99). The third period included any remaining studies in which data was collected on or before 31 July 2006 (i.e. before the Tinnitus Research Initiative (TRI) consensus meeting) [15], but not published until after this date (*n* = 27). Here we describe the patterns for the first two periods because of the comparable sample size and more robust definition, but all data are presented in Fig. 3. The Tinnitus Handicap Inventory, the Tinnitus Handicap Questionnaire, and the Tinnitus *Beeinträchtigungs Fragebogen* were equally popular across both 5-year periods. The Tinnitus Functional Index and numerical rating scales of tinnitus loudness increased in popularity, while the Tinnitus Questionnaire (German version), the Tinnitus Reaction Questionnaire, the Tinnitus Severity Index and tinnitus loudness matching all seemed to decrease in popularity.

### Pattern of usage of primary outcome instruments across interventions

All records were coded according to eight broad classes of procedure either as part of the intervention of interest or the control. These were: pharmacology (*n* = 66), electrophysiology (*n* = 59), sound therapy (*n* = 56), psychological therapy or counselling (referred to as ‘talking’) (*n* = 47), complementary therapy (*n* = 33), surgery (*n* = 10), manual physical therapy (*n* = 7), and relaxation (*n* = 3). Where interventions involved more than one procedure, all procedures were coded. For example, an intervention involving an intra-tympanic injection was coded as both pharmacology and surgery, and Tinnitus Retraining Therapy with a *Ginkgo biloba* supplement was coded as talking, sound and pharmacology.

The domain of tinnitus loudness was least frequently assessed in talking therapies (3 %), with the other major classes of intervention all assessing this perceptual characteristic more frequently: pharmacology (17 %), electrophysiology (16 %), sound therapy (15 %), and complementary therapy (14 %). In contrast, talking therapies favoured assessments of tinnitus distress (13 %) more than the other intervention classes: pharmacology (2 %), electrophysiology (6 %), sound therapy (7 %), and complementary therapy (4 %). Numerical rating scales and the Tinnitus Handicap Inventory were commonly used as outcome instruments for all types of interventions.

### Quality assessments

Following the protocol, we assessed the quality of defining and reporting outcomes in three ways. The first quality assessment considered the degree to which primary outcome instrument(s) in each study were appropriate and consistent with the authors’ choice of primary outcome domain(s). For example, the Tinnitus Severity Index would not be considered an ideal measure for quality of life, nor would ‘psychophysical method’ as a measure of tinnitus loudness. Within each study, we counted the number of consistent primary outcomes, calculated as a function of the proportion (%) of primary outcomes in that study. Overall, 31 (14 %) studies achieved a 100 % score, with 16 of those specifying a single primary outcome. In contrast to this, 133 (58 %) studies scored 0 %, with 52 of those failing to specify the primary domain and five not specifying the primary instrument. The remaining studies reported only partially correct outcomes: *n* = 5 scored 1–25 %, *n* = 31 scored 26–50 %, *n* = 21 scored 51–75 % and *n* = 7 scored 76–99 %.

The second quality assessment demonstrated that few trial designs were informed by a sample size calculation based on previous data for the primary outcome instrument. We excluded from this analysis 91 records that were trial registrations because a sample size calculation was not required for reporting. Of the remaining 137 records, sample size calculation was reported in only 37 of them (27 %). A sample size calculation requires specification of the primary outcome instrument, the expected difference between the treated and untreated groups, the pooled standard deviation, the desired statistical power, whether the hypothesis testing is one- or two-sided and the significance level (alpha). Over the 37 studies reporting sample size calculation, 31 (83 %) and 32 (86 %) studies reported statistical power and alpha value respectively, but the primary outcome instrument, the expected difference between groups and whether the test was one- or two-sided were mentioned in only 17 (46 %), 19 (51 %) and 14 (38 %) studies respectively. From the 17 studies reporting the primary outcome instrument, the Tinnitus Handicap Inventory was the most popular choice (*n* = 8). However, the magnitude of the expected change varied from study to study. It ranged from 6.55 to 20 points, but was also expressed as 50 % of reduction. Note that the developers of the Tinnitus Handicap Inventory recommend that a 20-point or greater change is required to account for test-retest variability [29].

**Fig. 4:**
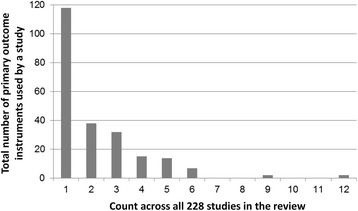
Number of primary outcome instruments reported across the 228 studies included in the review

The third quality assessment highlighted that many of the studies are suboptimal in terms of clearly defining what end point is the most important with respect to drawing a conclusion about treatment efficacy. For assessing whether an intervention has therapeutic benefit to patients, it is good practice to state a priori one outcome instrument [30]. Figure 4 illustrates the number of primary outcome instruments administered in each study. Just over half of all studies (118/228, 52 %) reported only one primary measure. However, the remainder reported multiple measures without distinguishing primary from secondary outcomes, with 70 studies (31 %) reporting two or three potential primary measures, 29 studies (13 %) reporting four or five and 11 studies reporting more than this. Two studies reported 12 measures without distinguishing primary from secondary outcomes [31, 32].

### Exploring the pattern of primary outcomes across tinnitus subgroups

A final analysis pre-specified in the published protocol was an exploratory one to address the question about whether a particular outcome domain (or instrument) was preferentially selected in trials enrolling a particular tinnitus subtype [16]. Here we considered tinnitus severity (as denoted by the authors), hearing loss, depression and anxiety because these are most relevant for determining choice of a tailored intervention.

#### Tinnitus severity

With respect to the primary domains, 96 out of the 505 came from studies that specified a severe tinnitus as an inclusion criterion. In those studies, an objective criterion was defined as some sort of minimum score on a published tinnitus questionnaire. For this subgroup compared to all 228 studies, we expected there would be a greater proportion of primary domains evaluating the functional impact of tinnitus, but this was not the case. The pattern was not noticeably different from the full dataset.

#### Hearing loss

Forty-seven of the 505 primary domains came from studies that specified a hearing loss as an inclusion criterion. Again, only studies were considered where an objective criterion had been defined and this was typically a minimum hearing level in dB at particular frequencies. Compared to the full dataset reported in Table 3, the proportion assessing tinnitus distress was slightly lower (4 % compared to 7 %). We also noted that the only study to report on a speech-based primary measure was part of this hearing loss subgroup [33]. Other audiological domains such as loudness and pitch had the same pattern of usage as the full dataset.

#### Depression and anxiety

Only one registered clinical trial actively recruited participants experiencing a comorbid depressive state [34], and no studies specified an inclusion criterion for a comorbid generalised anxiety. It is not possible, therefore, to consider any patterns within these subgroups.

## Discussion

There is a growing general recognition that insufficient attention has been paid to the outcomes measured in clinical trials [14]. Specifically, for tinnitus these limitations have been acknowledged in a number of systematic reviews, especially those published by the Cochrane Centre [35, 36], and have been highlighted by an international working party of the Tinnitus Research Initiative [15].

### Principal findings

No single outcome was reported in all studies. Instead a diverse range of outcomes were measured and reported. There are three key messages from our work.

First, over half of all studies did not adequately describe the domain for which they were predicting a predominant therapeutic benefit. In these cases, primary (and secondary) outcome domains were either not specified at all or were unclear. We believe that non-reporting mainly reflected a poor understanding of how important it is for individual trials to pre-specify the expected outcome. When conducting the review, we observed that the headings used within trial registries promote the reporting of instrument choice, rather than the outcome domain.

Second, there was extremely broad diversity of outcome instruments. Loudness was the most popular perceptual attribute of tinnitus described at the domain level, but there was no agreement on how to measure it and the precise methodology was often under-reported. Examples of descriptions for loudness matching included ‘matching at 1 kHz’, ‘psychoacoustical measure’, and ‘by audiometry’. Patient-reported questionnaires relating to the impact of tinnitus were the most common primary outcome instruments, but again there was no consensus about which one should be chosen. The Tinnitus Handicap Inventory remains the most popular questionnaire instrument simply because it is translated into the greatest number of languages. Certainly, it has limitations for the purpose of outcome measurement [28]. Worthy of note, we advise caution if pooling findings from the Tinnitus Handicap Inventory in a meta-analysis since it is unclear whether all translations achieve equivalence with the British original [37]. In compiling the list of tinnitus-related questionnaires (Table 4), it was striking how uninformative are the questionnaire names in helping trialists to choose between them. All include the word ‘tinnitus’ but rarely qualify that with a description of which tinnitus-related domains or constructs are assessed by the tool. Generic names and terms such as ‘handicap’ and ‘severity’ perpetuate the difficulty that many trialists experience in understanding what construct(s) a particular questionnaire instrument measures. For example, the Tinnitus Handicap Inventory [23] predominantly measures the construct of tinnitus-related distress, while the Tinnitus Handicap Questionnaire [38] measures the physical, emotional *and* social consequences of tinnitus, as well as hearing ability.

Third, treatment-related outcomes were rarely recorded. Safety, tolerability, side effects and withdrawals might be domains that all inform the measurement of adverse events, but these accounted for less than 2 % of primary outcome domains and 12 % of secondary outcome domains. Again, non-reporting mainly reflected a poor understanding of how important it is for individual trials to investigate and report harms, as well as benefits [39].

### Comparison with other studies

Our work provides the first detailed set of information on the selection and reporting of outcome domains and outcome instruments in clinical trials of tinnitus. One previous systematic review examined outcomes of randomised controlled trials of interventions for adults with tinnitus [11], but outcome data collection and reporting was restricted to ‘use of validated instruments for assessing tinnitus symptoms … any audiometric data … length of follow-up, and adverse event reporting.’ pp. 2–3, not the full set of outcomes considered in the present review. Reported findings indicated only that 20 % of studies used a validated tinnitus instrument, 79 % of studies used audiometric measurements, 42 % of studies specified adverse events, and the median follow-up time was 3 months. No further details were given and what constitutes a ‘validated instrument’ was not defined, so comparisons are restricted. Our study findings at least confirm the limited use of patient-reported questionnaire instruments relating to the impact of tinnitus. While we find little consistency across studies in reporting adverse events, our findings suggest that adverse event reporting is about 5 %, markedly less than the 42 % reported by Plein et al. [11].

Our review identifies limitations in the range of reported outcomes in clinical trials that are reflected more broadly across the field of audiological research. Here two reviews have been undertaken to identify outcome measures used in research on adults with hearing loss. In the first, Granberg et al. [40] conducted a systematic review of published articles, including a range of study designs. The authors found 51 different patient-reported questionnaire instruments relating to the impact of hearing loss out of the 122 studies included, with only 16 being used twice or more. Our review confirmed similar diversity (24 different tinnitus-related questionnaire instruments) and lack of consensus (14 used twice or more). In the second, Barker and et al. [41] conducted a scoping review to document the range and nature of outcome measurement in the context of adult auditory rehabilitation. Like us, they included registered trials and published studies. The most common outcome domain was ‘hearing handicap’ which was measured in 23 out of the 37 studies included, using five different patient-reported questionnaire instruments. Again, the use of generic terms such as ‘handicap’ perpetuate the difficulty that many trialists experience in understanding what construct(s) are measured by a particular questionnaire instrument. The frequency of reporting adverse events was not given by Granberg et al. [40], but Barker et al. [41] stated that no studies reported on adverse events. Poor reporting of harms-related data is not restricted to clinical trials in the hearing sciences [42].

### Strengths and limitations of the study

The strengths of our study rest on the inclusion of both registered (ongoing) clinical trials of tinnitus, as well as published study findings and on the broad-ranging and comprehensive evaluation of both the outcome domains and the outcome instruments used. Several potential limitations were unavoidable due to limited resources. These were the use of a pre-defined time window and the exclusion of non-English language records. While the search strategy excluded trials that were registered or published prior to July 2006, it is likely to have included trials designed prior to this date. However, there was insufficient information reported to ascertain this with any degree of certainty. Whether or not any systematic bias was introduced by the use of an English-language restriction is also uncertain, and may not affect systematic review conclusions [43].

Our study adds new insights to an emerging body of empirical evidence on outcome reporting within ENT and audiology trials [40, 41]. Our findings should help to steer trialists in these disciplines about good reporting practice, as well as to inform Cochrane and other systematic reviewers on the choice of outcomes for their work. Our study leads us to agree with Hoare et al. that ‘To be useful, future studies should … be consistent in their use of outcome measures’ [35].

The longer-term intention for this work is to develop a core outcome set that identifies by consensus a minimum standard for reporting in clinical trials of tinnitus in adults. This review makes a specific contribution to that ambitious endeavour by identifying which domains have been defined in relevant clinical trial designs to date. When developing a core outcome set, it is important to capture in the long list of potential outcome domains all those that need to be considered for inclusion [44]. For that long list to be truly comprehensive, it is important to capture relevant information that is contained within those studies. A limitation of the current review concerns those domain definitions that were unclear or not specified by their authors. This is especially important where the domains relate to patient-reported outcomes of the impact of tinnitus. One way to address the current gap is to deconstruct the patient-reported outcome instruments by creating a list of all questionnaire items, grouping individual items into similar constructs or domains and then cross-checking them against the current domain list reported here [44].

## Conclusions

We are the first group to conduct a systematic review that targets the reporting of outcome domains and instruments in clinical trial designs that evaluate interventions for tinnitus. The findings of this review have produced an extremely rich dataset that has enabled us to address a number of different primary and secondary questions concerning different aspects of good trial design. Our findings add important new insights pointing to the lack of awareness and understanding of good trial design in so far as this relates to outcomes. A general lack of consensus regarding the choice of outcomes did affect trial design, conduct and reporting with particular reference to lack of sample size calculation, and lack of robust interpretation of whether the intervention was therapeutically beneficial or not.

Our findings emphasise the need to improve trial design and reporting. A small number of the included studies in our review acknowledged Consolidated Standards of Reporting Trials (CONSORT) guidelines for reporting [45], but this is more the exception than the rule. Using such guidelines would improve definitions of all outcome measures including pre-specifying the time point of primary interest as well as detailed reporting of any important changes to methods or outcomes after the trial commenced with reasons for such changes. To improve reporting, we draw attention to the specialised CONSORT guidelines for reporting harms-related issues in a randomised controlled trial [39].

## Abbreviations

CDSR, Cochrane Database of Systematic Reviews; CENTRAL, Cochrane Central Register of Controlled Trials; CINAHL, Cumulative Index to Nursing and Allied Health Literature; CONSORT, Consolidated Standards of Reporting Trials; COST, Cooperation in Science and Technology; EBSCO, Elton Bryson Stephens COmpany; EMBASE (Ovid), Excerpta Medica Database; EPOC, Effective Practice and Organization of Care; ICTRP, International Clinical Trials Registry Platform; ISRCTN, International Standard Randomised Controlled Trial Number registry; PICOS, Patient, Intervention, Comparison, Outcome, Setting; PRISMA, Preferred Reporting Items for Systematic reviews and Meta-analyses; PROSPERO, International Prospective Register of Systematic Reviews; PubMed, database maintained by the United States National Library of Medicine at the National Institutes of Health; QoL, quality of life; TINNET, TINnitus research NETwork; TQ, Tinnitus Questionnaire; WHO, World Health Organisation; WHOQOL-BREF, World Health Organisation Quality of Life (brief version)

## Additional files

Additional file 1: PRISMA checklist of items to include when reporting a systematic review. (DOCX 19 kb) Additional file 2: TINNET WG5 master file. An editable version of the data master file. (XLSX 313 kb) Additional file 3: Table S1. Table of records containing missing data that was queried to the corresponding author by email. (DOCX 21 kb) Additional file 4: Table S2. Table reporting the full reference list for all 228 included records. (DOCX 39 kb) Additional file 5: Table S3. Tabulation of the evaluation of outcome reporting bias. ‘✓’ denotes consistent reporting across publications; ‘✗’ denotes inconsistent reporting; ‘o’ denotes partial reporting whereby the instrument remains consistent but the time frame does not; ‘P-only’ denotes that the outcome was specified in the protocol, but not reported as a study finding; ‘F-only’ denotes that the outcome was not specified in the protocol, but was reported as a study finding. For P-only, we cannot distinguish cases where an outcome was measured and analysed but not reported, measured but not analysed or reported, or not measured. (DOCX 25 kb) Additional file 6: Table S4. Outcome instruments used only once either for primary or secondary outcomes. (DOCX 19 kb)
