# Supplementary material for: Systematic review of outcome domains and instruments used in clinical trials of tinnitus treatments in adults
Source: Trials. 2016 Jun 1;17:270. doi: 10.1186/s13063-016-1399-9 (PMC4888312; doi:10.1186/s13063-016-1399-9)
Supplement: Additional file 3: Table S1. — Table of records containing missing data that was queried to the corresponding author by email. (DOCX 21 kb) [file 13063_2016_1399_MOESM3_ESM.docx]

**Additional Table 1.** Table of records containing missing data that was queried to the corresponding author by email.

| **Reference for the study record** | **Query** | **Response** |
| --- | --- | --- |
| Albu S, Chirtes F. Intratympanic dexamethasone plus melatonin versus melatonin only in the treatment of unilateral acute idiopathic tinnitus. American Journal of Otolaryngology Head and Neck Medicine and Surgery. 2014;35:617-22. | Minimum age | confirmed recruitment age ≥18 |
| Bauer C, Brozoski T. Effect of Tinnitus Retraining Therapy on the loudness and annoyance of tinnitus: a controlled trial. Ear and Hearing. 2011;2:145-55. | Minimum age | confirmed recruitment age ≥18 |
| Bessman P, Heider T, Watten R, Watten V. The Tinnitus Intensive Therapy Habituation Program: a 2-year follow-up pilot study on subjective tinnitus. Rehabilitation Psychology. 2009;2:133-57. | Minimum age | not responded |
| David BJ, Naftali A, Katz A. Tinntrain: a multifactorial treatment for tinnitus using binaural beats. The Hearing Journal. 2010;11:25-8. | Minimum age | author could not be contacted |
| Gerami H, Saberi A, Nemati S, Kazemnejad E, Aghajananpour M*.* Effects of oxcarbazepine versus carbamazepine on tinnitus: a randomized double-blind placebo-controlled clinical trial. Iranian Journal of Neurology. 2012;3:106-10. | Minimum age | not responded |
| Kim BG, Kim DY, Kim SK, Kim JM, Baek SH, Moon IS. Comparison of the outcomes of repetitive transcranial magnetic stimulation to the ipsilateral and contralateral auditory cortex in unilateral tinnitus. Electromagnetic Biology and Medicine. 2014;3:211-5. | Minimum age | not responded |
| Hatanaka A, Ariizumi Y, Kitamura K. Pros and cons of tinnitus retraining therapy. Acta Oto-Laryngologica. 2008;128:365-368. | Minimum age | author could not confirm recruitment age |
| Heijneman K, Kleine E, Dijk P. A randomized double-blind crossover study of phase-shift sound therapy for tinnitus. Otolaryngol Head Neck Surg. 2012;2:308-15. | Minimum age | confirmed recruitment age ≥18 |
| Herraiz C, Diges I, Cobo P. Auditory discrimination therapy (ADT) for tinnitus management. Progress in Brain Research. 2007;166:467-71. | Minimum age | author could not be contacted |
| Kim HJ, Kim DY, Kim HI, Oh HS, Sim NS, Moon IS. Long-term effects of repetitive transcranial magnetic stimulation in unilateral tinnitus. Laryngoscope. 2014; 124(9):2155-60. | Minimum age | confirmed recruitment age ≥18 |
| Kleinjung T, Eichhammer P, Landgrebe M, Sand P, Hajak G, Steffens T, et al. Combined temporal and prefrontal transcranial magnetic stimulation for tinnitus treatment: a pilot study. Otolaryngology–Head and Neck Surgery. 2008;138:497-501. | Minimum age | confirmed recruitment age ≥18 |
| Kleinjung T, Steffens T, Landgrebe M, Vielsmeier V, Frank E, Hajak G, et al. Levodopa does not enhance the effect of low-frequency repetitive transcranial magnetic stimulation in tinnitus treatment. Otolaryngology–Head and Neck Surgery. 2009;140:92-5. | Minimum age | confirmed recruitment age ≥18 |
| Koizumi T, Nishimura T, Sakaguchi T, Okamoto M, Hosoi H. Estimation of factors influencing the results of tinnitus retraining therapy. Acta Oto-Laryngologica. 2009;562:40-5. | Minimum age | not responded |
| Korres S, Mountricha A, Balatsouras D, Maroudias N, Riga M, Xenelis I. Tinnitus Retraining Therapy (TRT): outcomes after one-year treatment. International Tinnitus Journal. 2010;16(1):55-9. | Minimum age | confirmed recruitment age ≥18 |
| Malouff J, Noble W, Schutte N, Bhullar N. The effectiveness of bibliotherapy in alleviating tinnitus-related distress. Journal of Psychosomatic Research. 2010;68:245-51. | Minimum age | confirmed recruitment age ≥18 |
| Martz EC, Becker B. A comparison of three psychoeducational group interventions for veterans with tinnitus. ClinicalTrials.gov Identifier: NCT02293512 | Sample size | confirmed sample size=60 refers to the RCT. |
| Mielczarek M, Olszewski J. Direct current stimulation of the ear in tinnitus treatment: a double-blind placebo-controlled study. Eur Arch Otorhinolaryngol. 2014;271:1815-22. | Minimum age | confirmed recruitment age ≥18 |
| Punte A, Vermeire K, Hofkens A, De Bodt M, De Ridder D, Van de Heyning P. Cochlear implantation as a durable tinnitus treatment in single-sided deafness. Cochlear Implants International. 2011;S1:S26-9. | Minimum age | confirmed recruitment age ≥18 |
| Rabau S. Effect of time shift of transcranial direct current stimulation (tDCS) for treatment of acute tinnitus. ClinicalTrials.gov Identifier: NCT01886729. | Sample size | confirmed sample size and power calculation |
| Rabau S. The effect of transcranial direct current stimulation (tDCS) in addition to Tinnitus Retraining Therapy (TRT) for treatment of chronic tinnitus patients. ClinicalTrials.gov Identifier: NCT02285803. | Sample size | confirmed sample size and power calculation |
| Ridder D, Vanneste S, Kovacs, Sunaert S, Menovsky T, van de Heyning P, et al. Transcranial magnetic stimulation and extradural electrodes implanted on secondary auditory cortex for tinnitus suppression. J Neurosurg. 2011;114:903-11. | Minimum age | confirmed recruitment age ≥18 |
| Rocha CB, Sanchez TG. Efficacy of myofascial trigger point deactivation for tinnitus control. Braz J Otorhinolaryngol. 2012;78(6):21-6. | Minimum age | confirmed recruitment age ≥18 |
| Rogha M, Rezvani M, Khodami AR. The effects of acupuncture on the inner ear originated tinnitus. J Res Med Sci. 2011;16(9):1217–23. | Minimum age | confirmed recruitment age ≥18 |
| Sadlier M, Stephens S.D.G, Kennedy V. Tinnitus rehabilitation: a mindfulness meditation cognitive behavioural therapy approach. The Journal of Laryngology & Otology. 2008;122:31-7. | Minimum age | confirmed recruitment age ≥18 |
| Sekiya Y, Takahashi M, Kabaya K, Murakami S, Yoshioka M. Using fractal music as sound therapy in TRT treatment. AudiologyOnline. 2013;Article #11623. | Minimum age | author could not confirm recruitment age |
| Choi SJ, Lee JB, Lim HJ, In SM, Kim JY, Bae KH, et al. Intratympanic Dexamethasone injection for refractory tinnitus: prospective placebo-controlled study. Laryngoscope. 2013;123:2817-22. | Minimum age | confirmed recruitment age ≥18 |
| Shekhawat SG, Searchfield G, Stinear C. Randomized trial of transcranial direct current stimulation and hearing aids for tinnitus management Neurorehabilitation and Neural Repair. 2014;5:410-19. | Minimum age | confirmed recruitment age ≥18 |
| Sidheshwar P, Niladri KM, Ravishankar N. Role of self-induced sound therapy: Bhramari Pranayama in tinnitus. Audiological Medicine*.* 2010;8:137-41. | Minimum age | confirmed recruitment age ≥18 |
| Lee SK, Chung H, Chung JH, Yeo SG, Park MS, Byun JY. Effectiveness of transcutaneous electrical stimulation for chronic tinnitus. Acta Oto-laryngologica. 2014;134(2):159-67. | Minimum age | confirmed recruitment age ≥18 |
| Teggi R, Bellini C, Piccioni LO, Palonta F, Bussi M*.* Transmeatal low-level laser therapy for chronic tinnitus with cochlear dysfunction. Audiology and Neurotology. 2009;14:115-20. | Minimum age | confirmed all participants were of ‘major age’ |
| Trotter MI, Donaldson I. Hearing aids and tinnitus therapy: a 25-year experience. The Journal of Laryngology & Otology. 2008;122:1052-6. | Minimum age | confirmed recruitment age ≥18 |
| Tyler R, Noble W, Coelho C, Ji H. Tinnitus Retraining Therapy: mixing point and total masking are equally effective. Ear and Hearing. 2015;5:588-94. | Minimum age | author did respond but unwilling to provide information about recruitment age |
