# Supplementary material for: Systematic review of outcome domains and instruments used in clinical trials of tinnitus treatments in adults
Source: Trials. 2016 Jun 1;17:270. doi: 10.1186/s13063-016-1399-9 (PMC4888312; doi:10.1186/s13063-016-1399-9)
Supplement: Additional file 4: Table S2. — Table reporting the full reference list for all 228 included records. (DOCX 39 kb) [file 13063_2016_1399_MOESM4_ESM.docx]

**Additional Table 2.** Table reporting the full reference list for all 228 included records.

| Abbott JA, Kaldo V, Klein B, Austin D, Hamilton C, Piterman L, et al . A cluster randomised trial of an internet-based intervention program for tinnitus distress in an industrial setting. Cogn Behav Ther. 2009;38(3):162-73. |
| --- |
| Albu S, Chirtes F. Intratympanic dexamethasone plus melatonin versus melatonin only in the treatment of unilateral acute idiopathic tinnitus. Am J Otolaryngol. 2014 Sep-Oct;35(5):617-22. |
| Albu S, Chirtes F. Intratympanic dexamethasone plus melatonin versus melatonin only in the treatment of unilateral acute idiopathic tinnitus. American Journal of Otolaryngology Head and Neck Medicine and Surgery 2014;35:617-22. |
| Almeida TA, Samelli AG, Mecca Fdel N, De Martino E, Paulino AM. Tinnitus sensation pre and post nutritional intervention in metabolic disorders. Pro Fono. 2009 Oct-Dec;21(4):291-7. |
| Amanda B, Manuela M, Antonia M, Claudio M, Gregorio B. Posturography measures and efficacy of different physical treatments in somatic tinnitus. Int Tinnitus J. 2010;16(1):44-50. |
| Anders M, Dvorakova J, Rathova L, Havrankova P, Pelcova P, Vaneckova M, et al. Efficacy of repetitive transcranial magnetic stimulation for the treatment of refractory chronic tinnitus: a randomized, placebo controlled study. Neuro Endocrinol Lett. 2010;31(2):238-49. |
| ANZCTR: ACTRN12613000213741, *and* ICTRP record. |
| ANZCTR: ACTRN12613000685718, *and* ICTRP record. |
| Aoki M, Wakaoka Y, Hayashi H, Kuze B, Mizuta K, Ito Y. Effect of lyophilized powder made from enzymolyzed honeybee larvae on tinnitus-related symptoms, hearing levels, and hypothalamus-pituitary-adrenal axis-related hormones. Ear Hear. 2012 May-Jun;33(3):430-6. |
| Argstatter H, Grapp M, Hutter E, Plinkert P, Bolay HV. Long-term effects of the "Heidelberg Model of Music Therapy" in patients with chronic tinnitus. Int J Clin Exp Med. 2012;5(4):273-88. |
| Argstatter H, Grapp M, Hutter E, Plinkert PK, Bolay HV. The effectiveness of neuro-music therapy according to the Heidelberg model compared to a single session of educational counseling as treatment for tinnitus: a controlled trial. J Psychosom Res. 2015 Mar;78(3):285-92, *and* ClinicalTrials.gov Identifier: NCT01845155. |
| Argstatter H, Grapp M, Plinkert PK, Bolay HV. Heidelberg Neuro-Music Therapy for chronic-tonal tinnitus - treatment outline and psychometric evaluation. Int Tinnitus J. 2012;17(1):31-41. |
| Bakhshaee M, Ghasemi M, Azarpazhooh M, Khadivi E, Rezaei S, Shakeri M, et al. Gabapentin effectiveness on the sensation of subjective idiopathic tinnitus: a pilot study. Eur Arch Otorhinolaryngol. 2008 May;265(5):525-30. |
| Bauer C, Brozoski T. Effect of Tinnitus Retraining Therapy on the loudness and annoyance of tinnitus: a controlled trial. Ear and Hearing. 2011;2:145-155. |
| Biesinger E, Kipman U, Schätz S, Langguth B. Qigong for the treatment of tinnitus: a prospective randomized controlled study. J Psychosom Res. 2010 Sep;69(3):299-304. |
| Bilici S, Yigit O, Taskin U, Gor AP, Yilmaz ED. Medium-term results of combined treatment with transcranial magnetic stimulation and antidepressant drug for chronic tinnitus. Eur Arch Otorhinolaryngol. 2015 Feb;272(2):337-43. |
| Brazilian Registry of Clinical Trials: RBR-2t9t7q, *and* ICTRP record. |
| Caffier PP, Haupt H, Scherer H, Mazurek B. Outcomes of long-term outpatient tinnitus-coping therapy: psychometric changes and value of tinnitus-control instruments. Ear Hear. 2006 Dec;27(6):619-27. |
| Canis M, Olzowy B, Welz C, Suckfüll M, Stelter K. Simvastatin and Ginkgo biloba in the treatment of subacute tinnitus: a retrospective study of 94 patients. Am J Otolaryngol. 2011 Jan-Feb;32(1):19-23. |
| Chinese Clinical Trial Registry: ChiCTR-TRC-13003276, *and* ICTRP record. |
| Choi SJ, Lee JB, Lim HJ, In SM, Kim JY, Bae KH, et al. Intratympanic dexamethasone injection for refractory tinnitus: prospective placebo-controlled study. Laryngoscope. 2013 Nov;123(11):2817-22. |
| Choi SJ, Lee JB, Lim HJ, In SM, Kim JY, Bae KH, ey al. Intratympanic dexamethasone injection for refractory tinnitus: prospective placebo-controlled study. Laryngoscope. 2013 Nov;123(11):2817-22. |
| Choy DS, Lipman RA, Tassi GP. Worldwide experience with sequential phase-shift sound cancellation treatment of predominant tone tinnitus. J Laryngol Otol. 2010 Apr;124(4):366-9. |
| Chung HK, Tsai CH, Lin YC, Chen JM, Tsou YA, Wang CY, et al. Effectiveness of theta-burst repetitive transcranial magnetic stimulation for treating chronic tinnitus. Audiol Neurootol. 2012;17(2):112-20. |
| Cima RF, Maes IH, Joore MA, Scheyen DJ, El Refaie A, Baguley DM, et al. Specialised treatment based on cognitive behaviour therapy versus usual care for tinnitus: a randomised controlled trial. Lancet. 2012 May 26;379(9830):1951-9. |
| ClinicalTrials.gov Identifier: NCT00394056. |
| ClinicalTrials.gov Identifier: NCT00555776. |
| ClinicalTrials.gov Identifier: NCT00567892. |
| ClinicalTrials.gov Identifier: NCT00578058. |
| ClinicalTrials.gov Identifier: NCT00724152. |
| ClinicalTrials.gov Identifier: NCT00730834. |
| ClinicalTrials.gov Identifier: NCT00739635. |
| ClinicalTrials.gov Identifier: NCT00772980. |
| ClinicalTrials.gov Identifier: NCT00827008. |
| ClinicalTrials.gov Identifier: NCT00833950. |
| ClinicalTrials.gov Identifier: NCT00841230. |
| ClinicalTrials.gov Identifier: NCT00878696. |
| ClinicalTrials.gov Identifier: NCT00926237. |
| ClinicalTrials.gov Identifier: NCT00955799. |
| ClinicalTrials.gov Identifier: NCT01015781. |
| ClinicalTrials.gov Identifier: NCT01066273. |
| ClinicalTrials.gov Identifier: NCT01093872. |
| ClinicalTrials.gov Identifier: NCT01104207. |
| ClinicalTrials.gov Identifier: NCT01129141. |
| ClinicalTrials.gov Identifier: NCT01174979. |
| ClinicalTrials.gov Identifier: NCT01205919. |
| ClinicalTrials.gov Identifier: NCT01273883. |
| ClinicalTrials.gov Identifier: NCT01302873. |
| ClinicalTrials.gov Identifier: NCT01378650. |
| ClinicalTrials.gov Identifier: NCT01407133. |
| ClinicalTrials.gov Identifier: NCT01412918. |
| ClinicalTrials.gov Identifier: NCT01458821. |
| ClinicalTrials.gov Identifier: NCT01480193. |
| ClinicalTrials.gov Identifier: NCT01487447. |
| ClinicalTrials.gov Identifier: NCT01566708. |
| ClinicalTrials.gov Identifier: NCT01575496. |
| ClinicalTrials.gov Identifier: NCT01663311. |
| ClinicalTrials.gov Identifier: NCT01663467. |
| ClinicalTrials.gov Identifier: NCT01803646. |
| ClinicalTrials.gov Identifier: NCT01886092. |
| ClinicalTrials.gov Identifier: NCT01886729. |
| ClinicalTrials.gov Identifier: NCT01907022. |
| ClinicalTrials.gov Identifier: NCT01927991. |
| ClinicalTrials.gov Identifier: NCT01929837. |
| ClinicalTrials.gov Identifier: NCT01944501. |
| ClinicalTrials.gov Identifier: NCT01965028. |
| ClinicalTrials.gov Identifier: NCT01969474. |
| ClinicalTrials.gov Identifier: NCT02040194. |
| ClinicalTrials.gov Identifier: NCT02053961. |
| ClinicalTrials.gov Identifier: NCT02059447. |
| ClinicalTrials.gov Identifier: NCT02071732. |
| ClinicalTrials.gov Identifier: NCT02088866. |
| ClinicalTrials.gov Identifier: NCT02199106. |
| ClinicalTrials.gov Identifier: NCT02266160. |
| ClinicalTrials.gov Identifier: NCT02269839. |
| ClinicalTrials.gov Identifier: NCT02285803. |
| ClinicalTrials.gov Identifier: NCT02290015. |
| ClinicalTrials.gov Identifier: NCT02293512 . |
| ClinicalTrials.gov Identifier: NCT02306447. |
| ClinicalTrials.gov Identifier: NCT02315508. |
| ClinicalTrials.gov Identifier: NCT02353650. |
| ClinicalTrials.gov Identifier: NCT02370810. |
| ClinicalTrials.gov Identifier: NCT02383147. |
| ClinicalTrials.gov Identifier:NCT00596531. |
| ClinicalTrials.gov Identifier:NCT00628316. |
| ClinicalTrials.gov Identifier:NCT00876720. |
| Coelho C, Figueiredo R, Frank E, Burger J, Schecklmann M, Landgrebe M, et al. Reduction of tinnitus severity by the centrally acting muscle relaxant cyclobenzaprine: an open-label pilot study. Audiol Neurootol. 2012;17(3):179-88. |
| Coelho C, Witt SA, Ji H, Hansen MR, Gantz B, Tyler R. Zinc to treat tinnitus in the elderly: a randomized placebo controlled crossover trial. Otol Neurotol. 2013 Aug;34(6):1146-54, *and* ClinicalTrials.gov Identifier: NCT00683644. |
| Davis PB, Paki B, Hanley PJ. Neuromonics Tinnitus Treatment: third clinical trial. Ear Hear. 2007 Apr;28(2):242-59. |
| de Azevedo AA, Langguth B, de Oliveira PM, Rodrigues Figueiredo R. Tinnitus treatment with piribedil guided by electrocochleography and acoustic otoemissions. Otol Neurotol. 2009 Aug;30(5):676-80, *and* ClinicalTrials.gov Identifier: NCT00591994. |
| De Ridder D, Vanneste S, Kovacs S, Sunaert S, Menovsky T, van de Heyning P, et al. Transcranial magnetic stimulation and extradural electrodes implanted on secondary auditory cortex for tinnitus suppression. J Neurosurg. 2011 Apr;114(4):903-11. |
| De Ridder D, Vanneste S. EEG Driven tDCS Versus Bifrontal tDCS for Tinnitus. Front Psychiatry. 2012 Sep 25;3:84. |
| Dehkordi MA, Abolbashari S, Taheri R, Einolghozati S. Efficacy of gabapentin on subjective idiopathic tinnitus: a randomized, double-blind, placebo-controlled trial. Ear Nose Throat J. 2011 Apr;90(4):150-8. |
| Dehkordi MA, Einolghozati S, Ghasemi SM, Abolbashari S, Meshkat M, Behzad H. Effect of low-level laser therapy in the treatment of cochlear tinnitus: a double-blind, placebo-controlled study. Ear Nose Throat J. 2015 Jan;94(1):32-6, *and* ClinicalTrials.gov Identifier: NCT01268449. |
| Dib GC, Kasse CA, Alves de Andrade T, Gurgel Testa JR, Cruz OL. Tinnitus treatment with Trazodone. Braz J Otorhinolaryngol. 2007 May-Jun;73(3):390-7. |
| Dohrmann K, Elbert T, Schlee W, Weisz N. Tuning the tinnitus percept by modification of synchronous brain activity. Restor Neurol Neurosci. 2007;25(3-4):371-8. |
| Dohrmann K, Weisz N, Schlee W, Hartmann T, Elbert T. Neurofeedback for treating tinnitus. Prog Brain Res. 2007;166:473-85. |
| dos Santos GM, Bento RF, de Medeiros IR, Oiticcica J, da Silva EC, Penteado S. The influence of sound generator associated with conventional amplification for tinnitus control: randomized blind clinical trial. Trends Hear. 2014 Jul 23;18, *and* ClinicalTrials.gov Identifier: NCT01857661. |
| EudraCT Number: 2006-002692-41, *and* ICTRP record. |
| EudraCT Number: 2008-002369-29, *and* ICTRP record. |
| EudraCT Number: 2008-005178-10, *and* ICTRP record. |
| EudraCT Number: 2009-018046-38, *and* ICTRP record. |
| Figueiredo RR, Langguth B, Mello de Oliveira P, Aparecida de Azevedo A. Tinnitus treatment with memantine. Otolaryngol Head Neck Surg. 2008 Apr;138(4):492-6. |
| Formby C, Scherer R; TRTT Study Group. Rationale for the tinnitus retraining therapy trial. Noise Health. 2013 Mar-Apr;15(63):134-42, *and* Scherer RW, Formby C, Gold S, Erdman S, Rodhe C, Carlson M, Shade D, Tucker M, Sensinger LM, Hughes G, Conley GS, Downey N, Eades C, Jylkka M, et al. Tinnitus Retraining Therapy Trial Research Group. The Tinnitus Retraining Therapy Trial (TRTT): study protocol for a randomized controlled trial. Trials. 2014 Oct 15;15:396, *and* ClinicalTrials.gov Identifier: NCT01177137. |
| Forogh B, Yazdi-Bahri SM, Ahadi T, Fereshtehnejad SM, Raissi GR. Comparison of two protocols of transcranial magnetic stimulation for treatment of chronic tinnitus: a randomized controlled clinical trial of burst repetitive versus high-frequency repetitive Transcranial Magnetic Stimulation. Neurol Sci. 2014 Feb;35(2):227-32, *and* IRCT registration number: IRCT201112218438N1, *and* ICTRP record. |
| Forti S, Costanzo S, Crocetti A, Pignataro L, Del Bo L, Ambrosetti U. Are results of tinnitus retraining therapy maintained over time? 18-month follow-up after completion of therapy. Audiol Neurootol. 2009;14(5):286-9. |
| Grapp M, Hutter E, Argstatter H, Plinkert PK, Bolay HV. Music therapy as an early intervention to prevent chronification of tinnitus. Int J Clin Exp Med. 2013 Aug 1;6(7):589-93. |
| Gudex C, Skellgaard PH, West T, Sørensen J. Effectiveness of a tinnitus management programme: a 2-year follow-up study. BMC Ear Nose Throat Disord. 2009 Jun 26;9:6. |
| Gungor A, Dogru S, Cincik H, Erkul E, Poyrazoglu E. Effectiveness of transmeatal low power laser irradiation for chronic tinnitus. J Laryngol Otol. 2008 May;122(5):447-51. |
| Hanley PJ, Davis PB, Paki B, Quinn SA, Bellekom SR. Treatment of tinnitus with a customized, dynamic acoustic neural stimulus: clinical outcomes in general private practice. Ann Otol Rhinol Laryngol. 2008 Nov;117(11):791-9. |
| Hauptmann C, Ströbel A, Williams M, Patel N, Wurzer H, von Stackelberg T,et al. Acoustic Coordinated Reset Neuromodulation in a Real Life Patient Population with Chronic Tonal Tinnitus. Biomed Res Int. 2015;2015:569052, *and* ClinicalTrials.gov Identifier: NCT01435317. |
| Heijneman KM, de Kleine E, van Dijk P. A randomized double-blind crossover study of phase-shift sound therapy for tinnitus. Otolaryngol Head Neck Surg. 2012 Aug;147(2):308-15. |
| Henry JA, Frederick M, Sell S, Griest S, Abrams H. Validation of a novel combination hearing aid and tinnitus therapy device. Ear Hear. 2015 Jan;36(1):42-52. |
| Henry JA, Loovis C, Montero M, Kaelin C, Anselmi KA, Coombs R, et al. Randomized clinical trial: group counseling based on tinnitus retraining therapy. J Rehabil Res Dev. 2007;44(1):21-32. |
| Henry JA, Zaugg TL, Myers PJ, Schmidt CJ, Griest S, Legro MW, et al. Pilot study to develop telehealth tinnitus management for persons with and without traumatic brain injury. J Rehabil Res Dev. 2012;49(7):1025-42. |
| Herraiz C, Diges I, Cobo P, Aparicio JM, Toledano A. Auditory discrimination training for tinnitus treatment: the effect of different paradigms. Eur Arch Otorhinolaryngol. 2010 Jul;267(7):1067-74. |
| Hesser H, Gustafsson T, Lundén C, Henrikson O, Fattahi K, Johnsson E, Zetterqvist Westin V,et al. A randomized controlled trial of Internet-delivered cognitive behavior therapy and acceptance and commitment therapy in the treatment of tinnitus. J Consult Clin Psychol. 2012 Aug;80(4):649-61. |
| Hesser H, Pereswetoff-Morath CE, Andersson G. Consequences of controlling background sounds: the effect of experiential avoidance on tinnitus interference. Rehabil Psychol. 2009 Nov;54(4):381-9. |
| Hoare DJ, Pierzycki RH, Thomas H, McAlpine D, Hall DA. Evaluation of the acoustic coordinated reset (CR®) neuromodulation therapy for tinnitus: study protocol for a double-blind randomized placebo-controlled trial. Trials. 2013 Jul 10;14:207, *and* ClinicalTrials.gov Identifier: NCT01541969. |
| Hoare DJ, Van Labeke N, McCormack A, Sereda M, Smith S, Al Taher H,et al. Gameplay as a source of intrinsic motivation in a randomized controlled trial of auditory training for tinnitus. PLoS One. 2014 Sep 12;9(9):e107430, *and* ClinicalTrials.gov Identifier: NCT02095262 |
| Hoekstra CE, Versnel H, Neggers SF, Niesten ME, van Zanten GA. Bilateral low-frequency repetitive transcranial magnetic stimulation of the auditory cortex in tinnitus patients is not effective: a randomised controlled trial. Audiol Neurootol. 2013;18(6):362-73, *and* ClinicalTrials.gov Identifier: NCT00668720. |
| Hurtuk A, Dome C, Holloman CH, Wolfe K, Welling DB, Dodson EE, et al. Melatonin: can it stop the ringing? Ann Otol Rhinol Laryngol. 2011 Jul;120(7):433-40. |
| Ino T, Odaguchi H, Wakasugi A, Oikawa T, Sano H, Okamoto M, et al. A randomized, double-blind, placebo-controlled clinical trial to evaluate the efficacy of hangekobokuto in adult patients with chronic tinnitus. J. Trad. Med. 2013;30(2):72-81. |
| Iranian Registry of Clinical Trials: IRCT138709121483N1, *and* ICTRP record. |
| Iranian Registry of Clinical Trials: IRCT138903061138N2, *and* ICTRP record. |
| Iranian Registry of Clinical Trials: IRCT201105185867N4, *and* ICPRP record. |
| Iranian Registry of Clinical Trials: IRCT2012101811157N1, *and* ICTRP record. |
| Iranian Registry of Clinical Trials: IRCT2013011212101N1, *and* ICTRP record. |
| Iranian Registry of Clinical Trials: IRCT201403189014N32, *and* ICTRP record. |
| Iranian Registry of Clinical Trials: IRCT201404191138N11, *and* ICTRP record. |
| Iranian Registry of Clinical Trials: IRCT2014082018871N1, *and* ICTRP record. |
| ISRCTN registry: ISRCTN03638520. |
| ISRCTN registry: ISRCTN17631678. |
| ISRCTN registry: ISRCTN38408464. |
| ISRCTN registry: ISRCTN68772788. |
| ISRCTN registry: ISRCTN69465849. |
| Jalali MM, Kousha A, Naghavi SE, Soleimani R, Banan R. The effects of alprazolam on tinnitus: a cross-over randomized clinical trial. Med Sci Monit. 2009 Nov;15(11):PI55-60. |
| Japan Primary Registries Network: JPRN-JapicCTI-111648, *and* ICTRP record. |
| Japan Primary Registries Network: JPRN-UMIN000001357, *and* ICTRP record. |
| Japan Primary Registries Network: JPRN-UMIN000004110, *and* ICTRP record. |
| Japan Primary Registries Network: JPRN-UMIN000004766, *and* ICTRP record. |
| Japan Primary Registries Network: JPRN-UMIN000008355, *and* ICTRP record. |
| Japan Primary Registries Network: JPRN-UMIN000011643, *and* ICTRP record. |
| Japan Primary Registries Network: JPRN-UMIN000013421, *and* ICTRP record. |
| Jasper K, Weise C, Conrad I, Andersson G, Hiller W, Kleinstäuber M. Internet-based guided self-help versus group cognitive behavioral therapy for chronic tinnitus: a randomized controlled trial. Psychother Psychosom. 2014;83(4):234-46 and Conrad I, Kleinstäuber M, Jasper K, Hiller W, Andersson G, Weise C. The changeability and predictive value of dysfunctional cognitions in cognitive behavior therapy for chronic tinnitus. Int J Behav Med. 2015 Apr;22(2):239-50, *and* ClinicalTrials.gov Identifier: NCT01205906. |
| Jeon SW, Kim KS, Nam HJ. Long-term effect of acupuncture for treatment of tinnitus: a randomized, patient- and assessor-blind, sham-acupuncture-controlled, pilot trial. J Altern Complement Med. 2012 Jul;18(7):693-9. |
| Kaldo V, Cars S, Rahnert M, Larsen HC, Andersson G. Use of a self-help book with weekly therapist contact to reduce tinnitus distress: a randomized controlled trial. J Psychosom Res. 2007 Aug;63(2):195-202. |
| Kaldo V, Levin S, Widarsson J, Buhrman M, Larsen HC, et al. Internet versus group cognitive-behavioral treatment of distress associated with tinnitus: a randomized controlled trial. Behav Ther. 2008 Dec;39(4):348-59. |
| Kim HJ, Kim DY, Kim HI, Oh HS, Sim NS, Moon IS. Long-term effects of repetitive transcranial magnetic stimulation in unilateral tinnitus. Laryngoscope. 2014 Sep;124(9):2155-60. |
| Kim NK, Lee DH, Lee JH, Oh YL, Yoon IH, Seo ES, et al. Bojungikgitang and banhabaekchulchonmatang in adult patients with tinnitus, a randomized, double-blind, three-arm, placebo-controlled trial--study protocol. Trials. 2010 Mar 28;11:34, *and* ISRCTN registry: ISRCTN23691284. |
| Kim NK, Lee DH, Lee JH, Oh YL, Yoon IH, Seo ES, et al. Bojungikgitang and banhabaekchulchonmatang in adult patients with tinnitus, a randomized, double-blind, three-arm, placebo-controlled trial--study protocol. Trials. 2010 Mar 28;11:34, and ISRCTN registry: ISRCTN23691284. |
| Kleinjung T, Eichhammer P, Landgrebe M, Sand P, Hajak G, Steffens T, et al. Combined temporal and prefrontal transcranial magnetic stimulation for tinnitus treatment: a pilot study. Otolaryngol Head Neck Surg. 2008 Apr;138(4):497-501 |
| Kleinjung T, Steffens T, Landgrebe M, Vielsmeier V, Frank E, Hajak G, et al. Levodopa does not enhance the effect of low-frequency repetitive transcranial magnetic stimulation in tinnitus treatment. Otolaryngol Head Neck Surg. 2009 Jan;140(1):92-5. |
| Kleinjung T, Steffens T, Sand P, Murthum T, Hajak G, Strutz J, et al. Which tinnitus patients benefit from transcranial magnetic stimulation? Otolaryngol Head Neck Surg. 2007 Oct;137(4):589-95. |
| Korres S, Mountricha A, Balatsouras D, Maroudias N, Riga M, Xenelis I. Tinnitus Retraining Therapy (TRT): outcomes after one-year treatment. Int Tinnitus J. 2010;16(1):55-9 |
| Kreuzer PM, Goetz M, Holl M, Schecklmann M, Landgrebe M, Staudinger S, et al . Mindfulness-and body-psychotherapy-based group treatment of chronic tinnitus: a randomized controlled pilot study. BMC Complement Altern Med. 2012 Nov 28;12:235. |
| Kreuzer PM, Landgrebe M, Resch M, Husser O, Schecklmann M, Geisreiter F, et al. Feasibility, safety and efficacy of transcutaneous vagus nerve stimulation in chronic tinnitus: an open pilot study. Brain Stimul. 2014 Sep-Oct;7(5):740-7, *and* ClinicalTrials.gov Identifier: NCT01176734. |
| Kreuzer PM, Landgrebe M, Schecklmann M, Poeppl TB, Vielsmeier V, Hajak G, et al. Can Temporal Repetitive Transcranial Magnetic Stimulation be Enhanced by Targeting Affective Components of Tinnitus with Frontal rTMS? A Randomized Controlled Pilot Trial. Front Syst Neurosci. 2011 Nov 4;5:88, *and* ClinicalTrials.gov Identifier: NCT01261949. |
| Krings JG, Wineland A, Kallogjeri D, Rodebaugh TL, Nicklaus J, Lenze EJ, et al . A novel treatment for tinnitus and tinnitus-related cognitive difficulties using computer-based cognitive training and D-cycloserine. JAMA Otolaryngol Head Neck Surg. 2015 Jan;141(1):18-26, *and* ClinicalTrials.gov Identifier: NCT01550796. |
| Landgrebe M, Binder H, Koller M, Eberl Y, Kleinjung T, Eichhammer P, et al. Design of a placebo-controlled, randomized study of the efficacy of repetitive transcranial magnetic stimulation for the treatment of chronic tinnitus. BMC Psychiatry. 2008 Apr 15;8:23, *and* ISRCTN registry: ISRCTN89848288. |
| Langguth B, Landgrebe M, Frank E, Schecklmann M, Sand PG, Vielsmeier V, et al. Efficacy of different protocols of transcranial magnetic stimulation for the treatment of tinnitus: Pooled analysis of two randomized controlled studies. World J Biol Psychiatry. 2014 May;15(4):276-85. |
| Latifpour DH, Grenner J, Sjödahl C. The effect of a new treatment based on somatosensory stimulation in a group of patients with somatically related tinnitus. Int Tinnitus J. 2009;15(1):94-9. |
| Lee SK, Chung H, Chung JH, Yeo SG, Park MS, Byun JY. Effectiveness of transcutaneous electrical stimulation for chronic tinnitus. Acta Otolaryngol. 2014 Feb;134(2):159-67. |
| Lehner A, Schecklmann M, Kreuzer PM, Poeppl TB, Rupprecht R, Langguth B. Comparing single-site with multisite rTMS for the treatment of chronic tinnitus - clinical effects and neuroscientific insights: study protocol for a randomized controlled trial. Trials. 2013 Aug 23;14:269 and Erratum in: Trials. 2014;5:148, *and* ClinicalTrials.gov Identifier: NCT01663324. |
| Lipman RI, Lipman SP. Phase-shift treatment for predominant tone tinnitus. Otolaryngol Head Neck Surg. 2007 May;136(5):763-8. |
| Lugli M, Romani R, Ponzi S, Bacciu S, Parmigiani S. The windowed sound therapy: a new empirical approach for an effectiv personalized treatment of tinnitus. Int Tinnitus J. 2009;15(1):51-61. |
| Mahboubi H, Ziai K, Djalilian HR. Customized web-based sound therapy for tinnitus. Int Tinnitus J. 2012;17(1):26-30. |
| Malouff JM, Noble W, Schutte NS, Bhullar N. The effectiveness of bibliotherapy in alleviating tinnitus-related distress. J Psychosom Res. 2010 Mar;68(3):245-51. |
| Mazurek B, Haupt H, Szczepek AJ, Sandmann J, Gross J, Klapp BF, et al. Evaluation of vardenafil for the treatment ofsubjective tinnitus: a controlled pilot study. J Negat Results Biomed. 2009 Feb 17;8:3, *and* ClinicalTrials.gov Identifier: NCT00666809. |
| McNeill C, Távora-Vieira D, Alnafjan F, Searchfield GD, Welch D. Tinnitus pitch, masking, and the effectiveness of hearing aids for tinnitus therapy. Int J Audiol. 2012 Dec;51(12):914-9. |
| Mennemeier M, Chelette KC, Allen S, Bartel TB, Triggs W, Kimbrell T, et al. Variable changes in PET activity before and after rTMS treatment for tinnitus. Laryngoscope. 2011 Apr;121(4):815-22. |
| Michiels S, De Hertogh W, Truijen S, Van de Heyning P. Physical therapy treatment in patients suffering from cervicogenic somatic tinnitus: study protocol for a randomized controlled trial. Trials. 2014 Jul 22;15:297 and ClinicalTrials.gov Identifier: NCT02016313. |
| Mielczarek M, Konopka W, Olszewski J. The application of direct current electrical stimulation of the ear and cervical spine kinesitherapy in tinnitus treatment. Auris Nasus Larynx. 2013 Feb;40(1):61-5. |
| Mielczarek M, Olszewski J. Direct current stimulation of the ear in tinnitus treatment: a double-blind placebo-controlled study. Eur Arch Otorhinolaryngol. 2014 Jun;271(6):1815-22. |
| Mirvakili A, Mehrparvar A, Mostaghaci M, Mollasadeghi A, Mirvakili M, Baradaranfar M, et al. Low level laser effect in treatment of patients with intractable tinnitus due to sensorineural hearing loss. J Lasers Med Sci. 2014 Spring;5(2):71-4. |
| Munhoes dos Santos Ferrari G, Sanchez TG, Bovino Pedalini ME. The efficacy of open molds in controlling tinnitus. Braz J Otorhinolaryngol. 2007 May-Jun;73(3):370-7. |
| Myers PJ, Griest S, Kaelin C, Legro MW, Schmidt CJ, Zaugg TL, et al. Development of a progressive audiologic tinnitus management program for Veterans with tinnitus. J Rehabil Res Dev. 2014;51(4):609-22, *and* ClinicalTrials.gov Identifier: NCT00371436. |
| Neri G, Baffa C, De Stefano A, Poliandri A, Kulamarva G, Di Giovanni P, et al. Management of tinnitus: oral treatment with melatonin and sulodexide. J Biol Regul Homeost Agents. 2009 Apr-Jun;23(2):103-10. |
| Netherlands Trial Register: NTR2001, *and* ICTRP record. |
| Netherlands Trial Register: NTR1293, *and* ICTRP record. |
| Newman CW, Sandridge SA. A comparison of benefit and economic value between two sound therapy tinnitus management options. J Am Acad Audiol. 2012 Feb;23(2):126-38. |
| Nyenhuis N, Zastrutzki S, Weise C, Jäger B, Kröner-Herwig B. The efficacy of minimal contact interventions for acute tinnitus: a randomised controlled study. Cogn Behav Ther. 2013;42(2):127-38. |
| Oishi N, Kanzaki S, Shinden S, Saito H, Inoue Y, Ogawa K. Effects of selective serotonin reuptake inhibitor on treating tinnitus in patients stratified for presence of depression or anxiety. Audiol Neurootol. 2010;15(3):187-93. |
| Oishi N, Shinden S, Kanzaki S, Saito H, Inoue Y, Ogawa K. Effects of tinnitus retraining therapy involving monaural noise generators. Eur Arch Otorhinolaryngol. 2013 Feb;270(2):443-8. |
| Olze H, Szczepek AJ, Haupt H, Zirke N, Graebel S, Mazurek B. The impact of cochlear implantation on tinnitus, stress and quality of life in postlingually deafened patients. Audiol Neurootol. 2012;17(1):2-11. |
| Olzowy B, Canis M, Hempel JM, Mazurek B, Suckfüll M. Effect of atorvastatin on progression of sensorineural hearing loss and tinnitus in the elderly: results of a prospective, randomized, double-blind clinical trial. Otol Neurotol. 2007 Jun;28(4):455-8. |
| Oz I, Arslan F, Hizal E, Erbek SH, Eryaman E, Senkal OA, et al. Effectiveness of the combined hearing and masking devices on the severity and perception of tinnitus: a randomized, controlled, double-blind study. ORL J Otorhinolaryngol Relat Spec. 2013;75(4):211-20. |
| Pandey S, Mahato NK, Navale R. Role of self-induced sound therapy: Bhramari Pranayama in tinnitus. Audiological Medicine. 2010;8(3):137-41. |
| Pantev C, Rudack C, Stein A, Wunderlich R, Engell A, Lau P, et al. Study protocol: Münster tinnitus randomized controlled clinical trial-2013 based on tailor-made notched music training (TMNMT). BMC Neurol. 2014 Mar 2;14:40. |
| Parazzini M, Del Bo L, Jastreboff M, Tognola G, Ravazzani P. Open ear hearing aids in tinnitus therapy: An efficacy comparison with sound generators. Int J Audiol. 2011 Aug;50(8):548-53. |
| Philippot P, Nef F, Clauw L, de Romrée M, Segal Z. A randomized controlled trial of mindfulness-based cognitive therapy for treating tinnitus. Clin Psychol Psychother. 2012 Sep;19(5):411-9. |
| Philippot P, Nef F, Clauw L, de Romrée M, Segal Z. A randomized controlled trial of mindfulness-based cognitive therapy for treating tinnitus. Clin Psychol Psychother. 2012 Sep;19(5):411-9. |
| Piccirillo JF, Finnell J, Vlahiotis A, Chole RA, Spitznagel E Jr. Relief of idiopathic subjective tinnitus: is gabapentin effective? Arch Otolaryngol Head Neck Surg. 2007 Apr;133(4):390-7. |
| Plewnia C, Vonthein R, Wasserka B, Arfeller C, Naumann A, Schraven SP, Plontke SK. Treatment of chronic tinnitus with theta burst stimulation: a randomized controlled trial. Neurology. 2012;78(21):1628-34 *and* Arfeller C, Vonthein R, Plontke SK, Plewnia C. Efficacy and safety of bilateral continuous theta burst stimulation (cTBS) for the treatment of chronic tinnitus: design of a three-armed randomized controlled trial. Trials. 2009;10:74, *and* ClinicalTrials.gov Identifier: NCT00518024. |
| Punte AK, Vermeire K, Hofkens A, De Bodt M, De Ridder D, Van de Heyning P. Cochlear implantation as a durable tinnitus treatment in single-sided deafness. Cochlear Implants Int. 2011 May;12 Suppl 1:S26-9. |
| Robinson SK, Viirre ES, Bailey KA, Kin. A Randomized Controlled Trial of Cognitive-Behavior Therapy for Tinnitus. International Tinnitus Journal. 2008; 14(5):119-126. |
| Rocha CB, Sanchez TG. Efficacy of myofascial trigger point deactivation for tinnitus control. Braz J Otorhinolaryngol. 2012 Dec;78(6):21-6, *and* ClinicalTrials.gov Identifier: NCT00999648, *and* ICTRP record. |
| Rogha M, Rezvani M, Khodami AR. The effects of acupuncture on the inner ear originated tinnitus. J Res Med Sci. 2011 Sep;16(9):1217-23, and Iranian Registry of Clinical Trials: IRCT201106036699N1, *and* ICTRP record. |
| Seydel C, Haupt H, Szczepek AJ, Klapp BF, Mazurek B. Long-term improvement in tinnitus after modified tinnitus retraining therapy enhanced by a variety of psychological approaches. Audiol Neurootol. 2010;15(2):69-80. |
| Sharma DK, Kaur S, Singh J, Kaur I. Role of acamprosate in sensorineural tinnitus. Indian J Pharmacol. 2012 Jan;44(1):93-6. |
| Shekhawat GS, Searchfield GD, Kobayashi K, Stinear CM. Prescription of hearing-aid output for tinnitus relief. Int J Audiol. 2013 Sep;52(9):617-25. |
| Shekhawat GS, Searchfield GD, Stinear CM. Randomized Trial of Transcranial Direct Current Stimulation and Hearing Aids for Tinnitus Management. Neurorehabil Neural Repair. 2013 Nov 8;28(5):410-419. |
| Shekhawat GS, Stinear CM, Searchfield GD. Transcranial direct current stimulation intensity and duration effects on tinnitus suppression. Neurorehabil Neural Repair. 2013 Feb;27(2):164-72. |
| Shi GX, Han LL, Liu LY, Li QQ, Liu CZ, Wang LP. Acupuncture at local and distant points for tinnitus: study protocol for a randomized controlled trial. Trials. 2012 Nov 23;13:224, *and* ISRCTN registry: ISRCTN29230777. |
| Shi GX, Han LL, Liu LY, Li QQ, Liu CZ, Wang LP. Acupuncture at local and distant points for tinnitus: study protocol for a randomized controlled trial. Trials. 2012 Nov 23;13:224, and ISRCTN registry: ISRCTN29230777. |
| Sönmez O, Külahlı I, Vural A, Sahin MI, Aydın M. The evaluation of ozone and betahistine in the treatment of tinnitus. Eur Arch Otorhinolaryngol. 2013 Jul;270(7):1999-2006. |
| Staecker H, Maxwell KS, Morris JR, van de Heyning P, Morawski K, Reintjes F, et al. Selecting appropriate dose regimens for AM-101 in the intratympanic treatment of acute inner ear tinnitus. Audiol Neurootol. 2015;20(3):172-82, *and* ClinicalTrials.gov Identifier: NCT01270282. |
| Suckfüll M, Althaus M, Ellers-Lenz B, Gebauer A, Görtelmeyer R, Jastreboff PJ,et al. A randomized, double-blind, placebo-controlled clinical trial to evaluate the efficacy and safety of neramexane in patients with moderate to severe subjective tinnitus. BMC Ear Nose Throat Disord. 2011 Jan 11;11:1. |
| Sutbas A, Yetiser S, Satar B, Akcam T, Karahatay S, Saglam K. Low-cholesterol diet and antilipid therapy in managing tinnitus and hearing loss in patients with noise-induced hearing loss and hyperlipidemia. Int Tinnitus J. 2007;13(2):143-9. |
| Sziklai I, Szilvássy J, Szilvássy Z. Tinnitus control by dopamine agonist pramipexole in presbycusis patients: a randomized, placebo-controlled, double-blind study. Laryngoscope. 2011 Apr;121(4):888-93. |
| Tass PA, Adamchic I, Freund HJ, von Stackelberg T, Hauptmann C. Counteracting tinnitus by acoustic coordinated reset neuromodulation. Restor Neurol Neurosci. 2012;30(2):137-59, *and* ClinicalTrials.gov Identifier: NCT00927121. |
| Távora-Vieira D, Eikelboom RH, Miller S. Neuromonics tinnitus treatment for patients with significant level of hearing loss: an adaptation of the protocol. Int J Audiol. 2011 Dec;50(12):881-6. |
| Teggi R, Bellini C, Piccioni LO, Palonta F, Bussi M. Transmeatal low-level laser therapy for chronic tinnitus with cochlear dysfunction. Audiol Neurootol. 2009;14(2):115-20 |
| Thabit MN, Fouad N, Shahat B, Youssif M. Combined central and peripheral stimulation for treatment of chronic tinnitus: a randomized pilot study. Neurorehabil Neural Repair. 2015 Mar-Apr;29(3):224-33. |
| Thabit MN, Fouad N, Shahat B, Youssif M. Combined central and peripheral stimulation for treatment of chronic tinnitus: a randomized pilot study. Neurorehabil Neural Repair. 2015 Mar-Apr;29(3):224-33. |
| Topak M, Sahin-Yilmaz A, Ozdoganoglu T, Yilmaz HB, Ozbay M, Kulekci M. Intratympanic methylprednisolone injections for subjective tinnitus. J Laryngol Otol. 2009 Nov;123(11):1221-5. |
| Trotter MI, Donaldson I. Hearing aids and tinnitus therapy: a 25-year experience. J Laryngol Otol. 2008 Oct;122(10):1052-6. |
| van de Heyning P, Muehlmeier G, Cox T, Lisowska G, Maier H, Morawski K, et al. Efficacy and safety of AM-101 in the treatment of acute inner ear tinnitus--a double-blind, randomized, placebo-controlled phase II study. Otol Neurotol. 2014 Apr;35(4):589-97, *and* ClinicalTrials.gov Identifier: NCT00860808 |
| Vanneste S, Azevedo A, De Ridder D. The effect of naltrexone on the perception and distress in tinnitus: an open-label pilot study. Int J Clin Pharmacol Ther. 2013 Jan;51(1):5-11. |
| Vanneste S, Plazier M, Van de Heyning P, De Ridder D. Repetitive transcranial magnetic stimulation frequency dependent tinnitus improvement by double cone coil prefrontal stimulation. J Neurol Neurosurg Psychiatry. 2011 Oct;82(10):1160-4. |
| Vanneste S, Plazier M, Van de Heyning P, De Ridder D. Repetitive transcranial magnetic stimulation frequency dependent tinnitus improvement by double cone coil prefrontal stimulation. J Neurol Neurosurg Psychiatry. 2011 Oct;82(10):1160-4. |
| Wang K, Bugge J, Bugge S. A randomised, placebo-controlled trial of manual and electrical acupuncture for the treatment of tinnitus. Complement Ther Med. 2010 Dec;18(6):249-55. |
| Westin VZ, Schulin M, Hesser H, Karlsson M, Noe RZ, Olofsson U, et al . Acceptance and commitment therapy versus tinnitus retraining therapy in the treatment of tinnitus: a randomised controlled trial. Behav Res Ther. 2011 Nov;49(11):737-47. |
| Witsell DL, Hannley MT, Stinnet S, Tucci DL. Treatment of tinnitus with gabapentin: a pilot study. Otol Neurotol. 2007 Jan;28(1):11-5. |
| Xie H, Li X, Lai J, Zhou Y, Wang C, Liang J. Effectiveness of De Qi during acupuncture for the treatment of tinnitus: study protocol for a randomized controlled trial. Trials. 2014 Oct 15;15:397, *and* Chinese Clinical Trial Registry: ChiCTR-TRC-14004720. |
| Yazici ZM, Sayin I, Gökkuş G, Alatas E, Kaya H, Kayhan FT. Effectiveness of Ericksonian hypnosis in tinnitus therapy: preliminary results. B-ENT. 2012;8(1):7-12. |
| Yıldırım G, Berkiten G, Uğraş H, Saltürk Z.Changes in audiometry results following laser therapy for tinnitus. Eur J Gen Med 2011;8(4):284-290. |
| Zöger S, Erlandsson S, Svedlund J, HolgersK-M. Benefits from group psychotherapy in the treatment of severe refractory tinnitus. Audiological Medicine.2008;6(1):62-72. |
