# Supplementary material for: Systematic review of outcome domains and instruments used in clinical trials of tinnitus treatments in adults
Source: Trials. 2016 Jun 1;17:270. doi: 10.1186/s13063-016-1399-9 (PMC4888312; doi:10.1186/s13063-016-1399-9)
Supplement: Additional file 5: Table S3. — Tabulation of the evaluation of outcome reporting bias. ‘✓’ denotes consistent reporting across publications; ‘✗’ denotes inconsistent reporting; ‘o’ denotes partial reporting whereby the instrument remains consistent but the time frame does not; ‘P-only’ denotes that the outcome was specified in the protocol, but not reported as a study finding; ‘F-only’ denotes that the outcome was not specified in the protocol, but was reported as a study finding. For P-only, we cannot distinguish cases where an outcome was measured and analysed but not reported, measured but not analysed or reported, or not measured. (DOCX 25 kb) [file 13063_2016_1399_MOESM5_ESM.docx]

**Additional Table 3.** Tabulation of the evaluation of outcome reporting bias. ‘✓’ denotes consistent reporting across publications; ‘🗶’ denotes inconsistent reporting; ‘o’ denotes partial reporting whereby the instrument remains consistent but the time frame does not; ‘P-only’ denotes outcome was specified in the protocol, but not reported as a study finding; ‘F-only’ denotes outcome was not specified in the protocol, but was reported as a study finding. For P-only, we cannot distinguish cases where an outcome was measured and analysed but not reported, measured but not analysed or reported, or not measured.

| **Record code** | **Differences found between registered protocol and published results** | | | **Prospective registration** | |
| --- | --- | --- | --- | --- | --- |
|  | Inclusion/  exclusion criteria | Primary outcome | Secondary outcome |  |  |
| Plewnia C, Vonthein R, Wasserka B, Arfeller C, Naumann A, Schraven SP, Plontke SK. Treatment of chronic tinnitus with theta burst stimulation: a randomized controlled trial. Neurology. 2012;78(21):1628-34 *and* Arfeller C, Vonthein R, Plontke SK, Plewnia C. Efficacy and safety of bilateral continuous theta burst stimulation (cTBS) for the treatment of chronic tinnitus: design of a three-armed randomized controlled trial. Trials. 2009;10:74 *and* ClinicalTrials.gov Identifier: NCT00518024. | 🗶 | ✓ | 🗶 P-only  *and*  🗶 F-only | Y |  |
| de Azevedo AA, Langguth B, de Oliveira PM, Rodrigues Figueiredo R. Tinnitus treatment with piribedil guided by electrocochleography and acoustic otoemissions. Otol Neurotol. 2009 Aug;30(5):676-80 *and* ClinicalTrials.gov Identifier: NCT00591994. | 🗶 | o | o *and*  🗶 F-only | Y |  |
| Tass PA, Adamchic I, Freund HJ, von Stackelberg T, Hauptmann C. Counteracting tinnitus by acoustic coordinated reset neuromodulation. Restor Neurol Neurosci. 2012;30(2):137-59 *and* ClinicalTrials.gov Identifier: NCT00927121. | 🗶 | o | 🗶 F-only | Y |  |
| Forogh B, Yazdi-Bahri SM, Ahadi T, Fereshtehnejad SM, Raissi GR. Comparison of two protocols of transcranial magnetic stimulation for treatment of chronic tinnitus: a randomized controlled clinical trial of burst repetitive versus high-frequency repetitive Transcranial Magnetic Stimulation. Neurol Sci. 2014  Feb;35(2):227-32 *and* IRCT registration number: IRCT201112218438N1 *and* ICTRP record. | ✓ | 🗶 P-only | ✓ | N |  |
| Hoare DJ, Pierzycki RH, Thomas H, McAlpine D, Hall DA. Evaluation of the acoustic coordinated reset (CR®) neuromodulation therapy for tinnitus: study protocol for a double-blind randomized placebo-controlled trial. Trials. 2013 Jul 10;14:207 *and* ClinicalTrials.gov Identifier: NCT01541969 (with Study Results). | ✓ | o | o | Y |  |
| Kreuzer PM, Landgrebe M, Schecklmann M, Poeppl TB, Vielsmeier V, Hajak G, Kleinjung T, Langguth B. Can Temporal Repetitive Transcranial Magnetic  Stimulation be Enhanced by Targeting Affective Components of Tinnitus with Frontal rTMS? A Randomized Controlled Pilot Trial. Front Syst Neurosci. 2011 Nov 4;5:88 and ClinicalTrials.gov Identifier:  NCT01261949. | 🗶 | ✓ | 🗶 P-only  *and*  🗶 F-only | N |  |
| van de Heyning P, Muehlmeier G, Cox T, Lisowska G, Maier H, Morawski K, Meyer T. Efficacy and safety of AM-101 in the treatment of acute inner ear tinnitus--a double-blind, randomized, placebo-controlled phase II study. Otol Neurotol. 2014 Apr;35(4):589-97 *and* ClinicalTrials.gov Identifier: NCT00860808. | ✓ | 🗶 F-only | 🗶 F-only | Y |  |
| Mazurek B, Haupt H, Szczepek AJ, Sandmann J, Gross J, Klapp BF, Kiesewetter H, Kalus U, Stöver T, Caffier PP. Evaluation of vardenafil for the treatment of  subjective tinnitus: a controlled pilot study. J Negat Results Biomed. 2009 Feb 17;8:3 *and* ClinicalTrials.gov Identifier: NCT00666809. | 🗶 | ✓ | ✓ | Y |  |
| Hoekstra CE, Versnel H, Neggers SF, Niesten ME, van Zanten GA. Bilateral low-frequency repetitive transcranial magnetic stimulation of the auditory cortex  in tinnitus patients is not effective: a randomised controlled trial. Audiol Neurootol. 2013;18(6):362-73 *and* ClinicalTrials.gov Identifier: NCT00668720. | ✓ | ✓ | 🗶 P-only  *and*  o | Y |  |
| Jasper K, Weise C, Conrad I, Andersson G, Hiller W, Kleinstäuber M. Internet-based guided self-help versus group cognitive behavioral therapy for chronic tinnitus: a randomized controlled trial. Psychother Psychosom.  2014;83(4):234-46 *and* Conrad I, Kleinstäuber M, Jasper K, Hiller W, Andersson G, Weise C. The  changeability and predictive value of dysfunctional cognitions in cognitive behavior therapy for chronic tinnitus. Int J Behav Med. 2015 Apr;22(2):239-50 *and* ClinicalTrials.gov Identifier: NCT01205906. | 🗶 | 🗶 F-only *and*  o | 🗶 P-only  *and*  o | N |  |
| Dehkordi MA, Einolghozati S, Ghasemi SM, Abolbashari S, Meshkat M, Behzad H. Effect of low-level laser therapy in the treatment of cochlear tinnitus: a double-blind, placebo-controlled study. Ear Nose Throat J. 2015 Jan;94(1):32-6 *and* ClinicalTrials.gov Identifier:  NCT01268449 | 🗶 | 🗶 F-only | 🗶 F-only | N |  |
| Staecker H, Maxwell KS, Morris JR, van de Heyning P, Morawski K, Reintjes F, Meyer T. Selecting appropriate dose regimens for AM-101 in the intratympanic  treatment of acute inner ear tinnitus. Audiol Neurootol. 2015;20(3):172-82 *and* ClinicalTrials.gov Identifier:  NCT01270282. | 🗶 | ✓ | o | Y |  |
| Hauptmann C, Ströbel A, Williams M, Patel N, Wurzer H, von Stackelberg T, Brinkmann U, Langguth B, Tass PA. Acoustic Coordinated Reset Neuromodulation in a  Real Life Patient Population with Chronic Tonal Tinnitus. Biomed Res Int. 2015;2015:569052 *and* ClinicalTrials.gov Identifier: NCT01435317. | ✓ | ✓ | 🗶 P-only  *and*  🗶 F-only  *and*  o | Y |  |
| Argstatter H, Grapp M, Hutter E, Plinkert PK, Bolay HV. The effectiveness of neuro-music therapy according to the Heidelberg model compared to a single session of educational counseling as treatment for tinnitus: a controlled trial. J Psychosom Res. 2015 Mar;78(3):285-92 *and* ClinicalTrials.gov Identifier: NCT01845155. | 🗶 | o | 🗶 P-only | N |  |
| Kreuzer PM, Landgrebe M, Resch M, Husser O, Schecklmann M, Geisreiter F, Poeppl TB, Prasser SJ, Hajak G, Rupprecht R, Langguth B. Feasibility, safety and efficacy of transcutaneous vagus nerve stimulation in chronic tinnitus: an open pilot study. Brain Stimul. 2014 Sep-Oct;7(5):740-7 *and* ClinicalTrials.gov Identifier: NCT01176734. | ✓ | ✓ | 🗶 F-only | Y |  |
| Krings JG, Wineland A, Kallogjeri D, Rodebaugh TL, Nicklaus J, Lenze EJ, Piccirillo JF. A novel treatment for tinnitus and tinnitus-related cognitive difficulties using computer-based cognitive training and D-cycloserine. JAMA Otolaryngol Head Neck Surg. 2015 Jan;141(1):18-26 *and* ClinicalTrials.gov Identifier: NCT01550796. | 🗶 | 🗶 F-only | 🗶 F-only | Y |  |
| Myers PJ, Griest S, Kaelin C, Legro MW, Schmidt CJ, Zaugg TL, Henry JA. Development of a progressive audiologic tinnitus management program for Veterans  with tinnitus. J Rehabil Res Dev. 2014;51(4):609-22 *and* ClinicalTrials.gov Identifier: NCT00371436. | 🗶 | ✓ | 🗶 P-only | Y |  |
| Coelho C, Witt SA, Ji H, Hansen MR, Gantz B, Tyler R. Zinc to treat tinnitus in the elderly: a randomized placebo controlled crossover trial. Otol Neurotol. 2013 Aug;34(6):1146-54 and ClinicalTrials.gov Identifier:  NCT00683644. | ✓ | 🗶 P-only  *and*  🗶 F-only  *and*  o | 🗶 P-only  *and*  🗶 F-only  *and*  o | Y |  |
| Mollasadeghi A, Mirmohammadi SJ, Mehrparvar AH, Davari MH, Shokouh P, Mostaghaci M, Baradaranfar MH, Bahaloo M. Efficacy of low-level laser therapy in  the management of tinnitus due to noise-induced hearing loss: a double-blind randomized clinical trial. Scientific World Journal. 2013 Oct 28;2013:596076 and  ANZCTR: ACTRN12612000455864 *and* ICTRP record. | ✓ | 🗶 F-only | ✓ | N |  |
| Rogha M, Rezvani M, Khodami AR. The effects of acupuncture on the inner ear originated tinnitus. J Res Med Sci. 2011 Sep;16(9):1217-23 *and* IRCT registration number : IRCT201106036699N1 *and* ICTRP record. | ✓ | ✓ | 🗶 F-only | N |  |
| Rocha CB, Sanchez TG. Efficacy of myofascial trigger point deactivation for tinnitus control. Braz J Otorhinolaryngol. 2012 Dec;78(6):21-6 *and* ClinicalTrials.gov Identifier: NCT00999648 *and* ICTRP record. | ✓ | o | o | N |  |
