# Supplementary material for: Systematic review of outcome domains and instruments used in clinical trials of tinnitus treatments in adults
Source: Trials. 2016 Jun 1;17:270. doi: 10.1186/s13063-016-1399-9 (PMC4888312; doi:10.1186/s13063-016-1399-9)
Supplement: Additional file 6: Table S4. — Outcome instruments used only once either for primary or secondary outcomes. (DOCX 19 kb) [file 13063_2016_1399_MOESM6_ESM.docx]

**Additional Table 4.** Outcome instruments used only once either for primary or secondary outcomes.

|  | **Primary outcome instruments** | | **Secondary outcome instruments** | |
| --- | --- | --- | --- | --- |
|  | **Number** | **Percentage (%)** | **Number** | **Percentage (%)** |
| ***(1) Investigator-administered tests relating to the tinnitus percept*** | | |  |  |
| Acufenometry evaluation | 1 | 0% | 0 | - |
| Phase-out treatment device (loudness measure) | 1 | 0% | 0 | - |
| Computerised tinnitus loudness assessment | 1 | 0% | 0 | - |
| ***(2) Patient-reported questionnaire instruments relating to the impact of tinnitus*** | | | |  |
| Klockhoff Self-Evaluation | 1 | 0% | 0 | - |
| Tinnitus and Hearing Survey | 1 | 0% | 1 | 0% |
| Tinnitus Coping Style Questionnaire | 1 | 0% | 1 | 0% |
| Tinnitus Handicap Inventory-12 | 1 | 0% | 0 | - |
| Tinnitus Loudness Questionnaire | 1 | 0% | 0 | - |
| Tinnitus Questionnaire (Persian version) | 1 | 0% | 0 | - |
| Tinnitus Catastrophy Scale | 0 | - | 1 | 0% |
| Tinnitus Cognitions Questionnaire | 0 | - | 1 | 0% |
| Tinnitus Cognitions Scale | 0 | - | 1 | 0% |
| Tinnitus Fear Avoidance Scale | 0 | - | 1 | 0% |
| ***(3) Patient-reported questionnaire instruments relating to other co-occurring complaints*** | | | | |
| General Depression Scale | 1 | 0% | 0 | - |
| General Health Questionnaire-12 | 1 | 0% | 0 | - |
| Hamilton Depression Scale | 1 | 0% | 0 | - |
| Hamilton Anxiety Scale | 0 | - | 1 | 0% |
| Hyperacusis questionnaire (adapted from Dauman and Bouscau-Faure, 2005) | 1 | 0% | 0 | - |
| Hyperacusis Questionnaire (GÜF) | 1 | 0% | 1 | 0% |
| Modified Somatic Perception Questionnaire | 1 | 0% | 0 | - |
| Neck Bournemouth Questionnaire | 1 | 0% | 0 | - |
| Private Self-consciousness Scale | 1 | 0% | 0 | - |
| Symptom Checklist 90, revised (SCL-90-R) | 1 | 0% | 0 | - |
| Vital exhaustion questionnaire | 1 | 0% | 1 | 0% |
| Anxiety Sensitivity Index | 0 | - | 1 | 0% |
| Auditory Hypersensitivity Questionnaire | 0 | - | 1 | 0% |
| Beck Anxiety Inventory | 0 | - | 1 | 0% |
| Mindful Attention Awareness Scale | 0 | - | 1 | 0% |
| Neck Pain and Disability Scale | 0 | - | 1 | 0% |
| Occupational Stress Inventory | 0 | - | 1 | 0% |
| Patient Health Questionnaire (PHQ-9) | 0 | - | 1 | 0% |
| Perceived Stress Scale | 0 | - | 1 | 0% |
| Profile of Mood States questionnaire | 0 | - | 1 | 0% |
| Psychiatric Signs Screening | 0 | - | 1 | 0% |
| Web Screening Questionnaire for Common Mental Disorders | 0 | - | 1 | 0% |
| Von Zerssen’s symptom list | 0 | - | 1 | 0% |
| ***(4) Patient-reported questionnaire instruments relating to health-related quality of life*** | | | | |
| Health Utilities Index mark 3 | 1 | 0% | 1 | 0% |
| Nijmegen Cochlear Implantation Questionnaire | 1 | 0% | 0 | - |
| Quality of Well-Being Scale | 1 | 0% | 0 | - |
| Satisfaction with Life Scale | 1 | 0% | 0 | - |
| Sheehan Disability Scale | 1 | 0% | 1 | 0% |
| Clinical Outcomes in Routine Evaluation | 0 | - | 1 | 0% |
| Personal Resources Questionnaire | 0 | - | 1 | 0% |
| 12-item short form Health Survey | 0 | - | 1 | 0% |
| Social Support Questionnaire | 0 | - | 1 | 0% |
| Work and Social Adjustments Scale | 0 | - | 1 | 0% |
| ***(5) Technical and laboratory measurements relating to body structure and function*** | | | | |
| Blood pressure | 1 | 0% | 1 | 0% |
| Flexion-extension, Lateral flexion, Rotation measured by Myrin | 1 | 0% | 0 | - |
| Heart rate | 1 | 0% | 1 | 0% |
| Residual Inhibition | 1 | 0% | 1 | 0% |
| Thoracic kyphosis, Lumbar lordosis measured by Debrunner’s kyphometer | 1 | 0% | 0 | - |
| Body weight | 0 | - | 1 | 0% |
| Electrocochleography | 0 | - | 1 | 0% |
| Glucose Tolerance Test | 0 | - | 1 | 0% |
| Otoacoustic Emissions | 0 | - | 1 | 0% |
| Paced Auditory Serial Addition Test | 0 | - | 1 | 0% |
| Persistent eardrum perforation | 0 | - | 1 | 0% |
| Posturography oscillation measure | 0 | - | 1 | 0% |
| Saliva biomarkers | 0 | - | 1 | 0% |
| Skin temperature and conductance | 0 | - | 1 | 0% |
| Spontaneous nystagmus | 0 | - | 1 | 0% |
| Stroop color word test | 0 | - | 1 | 0% |
| Test of Everyday Attention | 0 | - | 1 | 0% |
| Transcranial Magnetic Stimulation | 0 | - | 1 | 0% |
| ***(6) Measures of adverse events or harms*** | No instruments reported | | | |
| ***(7) Measures of satisfaction*** | No instruments reported | | | |
| ***(8) Measurement instruments of treatment-related outcomes*** | | | | |
| Massachusetts General Hospital acupuncture sensation scale (Chinese version) | 0 | - | 1 | 0% |
| Treatment Credibility Scale | 0 | - | 1 | 0% |
| Working Alliance Inventory (German version) | 0 | - | 1 | 0% |
| ***(9) Other (purpose of measurement not specified by the authors)*** | | | | |
| Eysenck Personality Questionnaire | 0 | - | 1 | 0% |
| Type D Personality Scale | 0 | - | 1 | 0% |
| numerical rating scale of tinnitus handicap | 1 | 0% | 1 | 0% |
| numerical rating scale (miscellaneous) | 26 | 5% | 14 | 2% |
